# The *pch2Δ* Mutation in Baker's Yeast Alters Meiotic Crossover Levels and Confers a Defect in Crossover Interference

**DOI:** 10.1371/journal.pgen.1000571

**Published:** 2009-07-24

**Authors:** Sarah Zanders, Eric Alani

**Affiliations:** Department of Molecular Biology and Genetics, Cornell University, Ithaca, New York, United States of America; The University of North Carolina at Chapel Hill, United States of America

## Abstract

Pch2 is a widely conserved protein that is required in baker's yeast for the organization of meiotic chromosome axes into specific domains. We provide four lines of evidence suggesting that it regulates the formation and distribution of crossover events required to promote chromosome segregation at Meiosis I. First, *pch2Δ* mutants display wild-type crossover levels on a small (III) chromosome, but increased levels on larger (VII, VIII, XV) chromosomes. Second, *pch2Δ* mutants show defects in crossover interference. Third, crossovers observed in *pch2Δ* require both Msh4-Msh5 and Mms4-Mus81 functions. Lastly, the *pch2Δ* mutation decreases spore viability and disrupts crossover interference in *spo11* hypomorph strains that have reduced levels of meiosis-induced double-strand breaks. Based on these and previous observations, we propose a model in which Pch2 functions at an early step in crossover control to ensure that every homolog pair receives an obligate crossover.

## Author Summary

During meiosis, cells that ultimately become gametes (such as eggs or sperm) undergo a single round of DNA replication followed by two consecutive divisions. In most organisms, the segregation of chromosomes at the first meiotic division is dependent upon genetic exchange, or crossing over, at homologous sites along chromosomes. Crossing over must therefore be regulated to ensure that every pair of matched chromosomes receives at least one crossover. Matched chromosomes that do not receive a crossover frequently undergo missegregation at the first meiotic division, yielding gametes that do not contain the normal chromosome number. Such missegregation events have been linked to human infertility syndromes. We used a genetic approach to study meiotic crossover control in baker's yeast. Our work suggests that Pch2 is required in crossover control during meiosis; mutants lacking Pch2 display altered crossover levels and distribution. Furthermore, *pch2* mutations cause enhanced gamete inviability in strains that are mildly defective in initiating recombination. Based on these observations, we hypothesize that Pch2 acts early in crossover control, in steps that occur prior to those proposed for previously characterized crossover-promoting factors.

## Introduction

Meiosis generates haploid gametes from diploid progenitor cells. The reduction in ploidy results from the segregation of homologous chromosome pairs in the first meiotic division (MI, [1]). Prior to MI, each chromosome is joined to its homolog at chiasmata, which serve to tether homologs to each other. This interaction promotes the tension between homologs needed to form a bipolar spindle that facilitates homolog segregation. Homologous chromosome pairs lacking chiasmata connections often fail to segregate properly at MI. Chromosome nondisjunction can also result if chiasmata are present, but not properly placed on chromosomes, or if sister chromatid cohesion is disrupted [2]–[5]. Regardless of the cause, chromosome missegregation produces aneuploid gametes that lead to infertility or conditions like Down syndrome in humans [6].

Chiasmata form at sites where programmed Spo11-catalyzed DNA double-stranded breaks (DSBs), induced early in meiotic prophase, are repaired to form crossovers [1]. In baker's yeast, crossovers (COs) are formed via two main pathways. The first pathway, by which the majority of COs are made, involves Msh4-Msh5 and Mlh1-Mlh3 [7]–[15]. In this pathway, DSBs are processed and acted upon by strand exchange enzymes to form single-end invasion intermediates (SEIs) that are converted into double Holliday junctions (dHJs). The latter are resolved into crossovers which display interference; the COs are more uniformly spaced than if placed at random (see below; [16]–[22]). The COs formed via the second major pathway, which require Mms4-Mus81, are not subject to CO interference [10],[11],[23]. Little is known about the intermediates that form in this latter pathway.

The recombination steps that lead to CO formation occur in meiotic prophase. In leptotene, when meiotic DSB formation initiates recombination, an axial element containing Hop1 and Red1 proteins assembles along each pair of sister chromatids. In zygotene, when SEIs are detected, mature tripartite synaptonemal complex (SC) starts to form when the Zip1-containing central element connects the axial elements, which are now termed “lateral elements.” Mature SC initiation begins at centromeres and later at CO-designated sites. These SC initiation events then spread outward until synapsis is completed in pachytene [24],[25]. Hop1/Red1 and Zip1 are enriched in separate domains on the mature SC. This organization is Pch2-dependent because in *pch2Δ* mutants, Zip1 and Hop1 appear to be more uniformly distributed along the chromosome axes [26],[27]. At the end of pachytene, recombination intermediates are resolved (reviewed in [28]).

In yeast, ∼40% of the ∼140–170 meiotic DSBs are repaired to generate noncrossover (NCO) products [29],[30]. These NCO products are thought to form by a synthesis-dependent strand annealing mechanism (SDSA, [31]), separate from the interfering CO mechanism, and do not result in MI disjunction-promoting chiasmata. Martini *et al.* [32] found that when meiotic programmed DSBs are decreased in *spo11* hypomorphic strains, COs are favored at the expense of NCOs [24]. This CO homeostasis phenomenon may be an additional manifestation of CO interference [32],[33]. The above studies indicate that DSBs are subject to a CO vs. NCO decision step, which is regulated by interference. Interference regulates this decision by ensuring that CO designation for a given DSB inhibits nearby DSBs from receiving this designation, thereby relegating them to a NCO fate. It is not clear whether non-interfering COs are formed through such a decision process; these COs are thought to form through a parallel pathway [10],[23]. For this paper, the CO vs. NCO decision refers solely to COs that are subject to interference.

The interference-regulated CO vs. NCO decision likely occurs very early in recombination, roughly at the time of SEI formation (late leptotene-early zygotene) and does not appear to be controlled by domains or sequences contained within the chromosome [17],[18],[20],[21],[34],[35]. CO interference is strongest near a CO event and weakens with distance along the chromosome, although interference can act over large distances, up to ∼150 kb in yeast and ∼60 Mb in mice [30],[33],[36],[37]. In addition, interference between COs appears stronger on longer chromosomes compared to shorter chromosomes [35], [38]–[40], but see [41]. However, smaller chromosomes have relatively high DSB density and may also have a higher density of non-interfering COs [39],[41],[42].

The mechanisms underlying interference regulation of the CO vs. NCO decision are unknown, despite the fact that numerous mutants showing defects in CO interference have been identified in baker's yeast. For at least a subset of these mutants, the defects in CO interference likely reflect problems in CO formation and not in the early CO vs. NCO decision (reviewed in [28]). For example, mutants defective in either the SC central element protein Zip1 or the CO-promoting factor Msh4 have reduced CO levels and the remaining COs show reduced or no interference [7], [33], [43]–[45]. However, Zip2 foci, which mark the early CO designated sites, still display interference in *zip1* and *msh4* mutants [35]. This result, combined with the fact that NCOs form in *zip1Δ* and *msh4Δ*, suggests that interference regulation of the CO vs. NCO decision requires neither these factors nor the mature SC [20],[22],[35],[46]. Rather, Zip1 and Msh4 are needed downstream of the decision to ensure CO formation [22],[35]. It is likely that these results are applicable to other members of the ZMM (Zip, Msh, Mer) class of proteins.

We examined the *PCH2* gene for a role in interference regulation of the CO vs. NCO decision. *PCH2* is a putative AAA-ATPase widely conserved in organisms that construct a synaptonemal complex in meiosis [26],[47]. *PCH2* was first identified in *S. cerevisiae* as a meiotic checkpoint factor due to the ability of *pch2Δ* to suppress the meiotic arrest of *zip1Δ* mutants [26]. This observation was extended by Wu and Burgess [47]; they proposed that Pch2 and Rad17 comprise separate branches of a checkpoint that ensures proper timing of the MI division, with the Pch2-dependent branch monitoring synaptonemal complex formation and the Rad17-dependent branch monitoring recombination events. Other checkpoint roles for Pch2 were reported in *C. elegans*, where it is required for apoptosis in response to unsynapsed pairing centers and in *Drosophila*, where it is required to delay meiotic progression in certain CO formation mutants [48],[49].

Recent studies indicate that *PCH2* is not solely a checkpoint factor; it is essential for proper meiotic axis organization and timely meiotic progression in baker's yeast, and complete DSB repair and fertility in mice [27],[47],[50]. Here we report that *pch2* mutants display increased meiotic CO levels on larger chromosomes and are defective in CO interference. We also show that mutation of *PCH2* reduces spore viability in *spo11* hypomorphic strains. These data support an early role for Pch2 in DSB repair and a model in which Pch2-promoted meiotic axis organization controls CO levels and their distribution.

## Results

### Genetic analysis of recombination

#### A new phenotype for *pch2Δ* mutants: increased crossing over on large chromosomes

**Figure 1:**
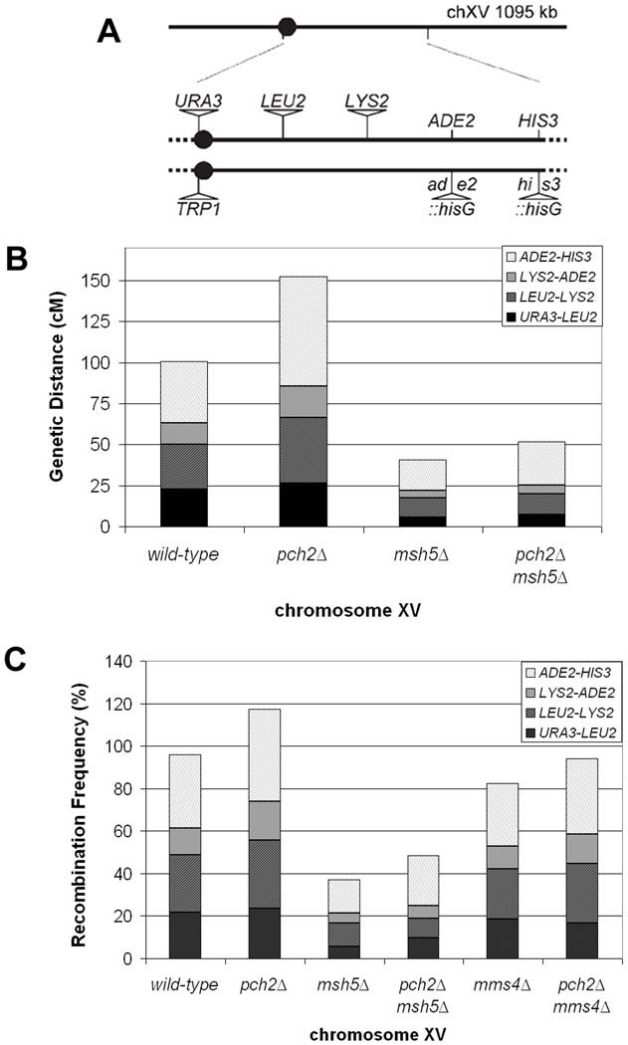
*pch2Δ* has increased levels of meiotic COs on the large chromosome XV. Recombination levels in four genetic intervals were analyzed on chromosome XV in the EAY1108/EAY1112 strain background (A). CO frequencies were calculated in cM from tetrads (B) and as recombination frequencies in spores (C). See Table 1 and Table S3 for raw data and statistical analyses.

**Figure 2:**
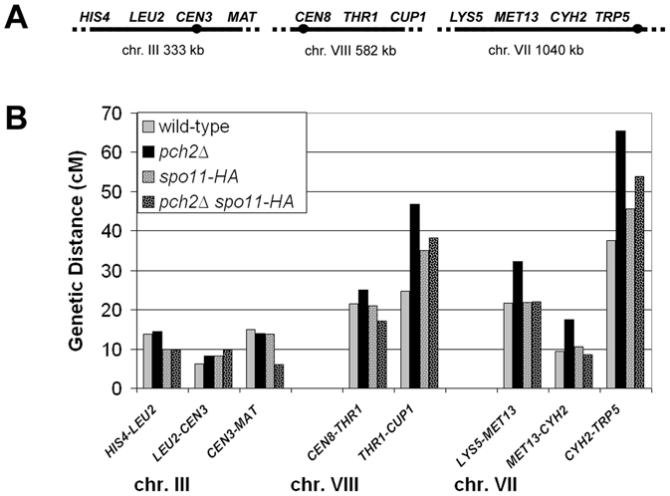
*pch2Δ* has increased meiotic CO levels on chromosomes VII and VIII, but not the small chromosome III. (A) The organization of genetic markers assayed on a small (III), medium (VIII), and large (VII) chromosome in the NHY942/NHY943 strain background is shown. (B) CO frequencies in cM were calculated from four-spore viable tetrads in wild-type, *pch2Δ/pch2Δ*, *spo11-HA/spo11-HA* and *pch2Δ spo11-HA/spo11-HA* strains. See Table 1 for raw data and statistical analyses.

**Figure 3:**
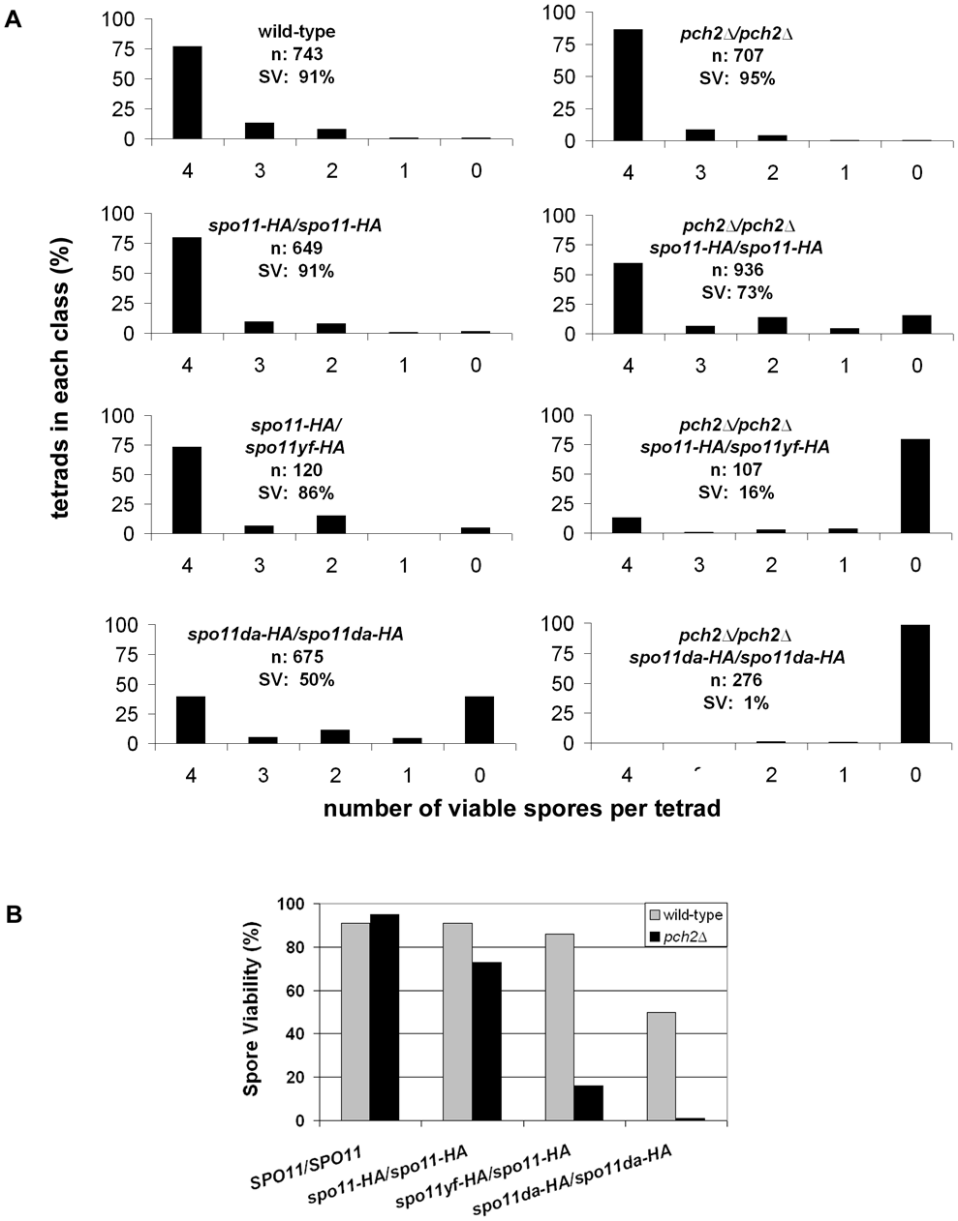
Pch2 promotes spore viability in *spo11* hypomorphs. (A) Spore viability distributions of wild-type and mutant strains in the NHY942/NHY943 strain background are displayed. The X-axes represent the number of viable spores per tetrad and the Y-axes represent the percent of tetrads comprising each class. The total number of tetrads dissected (n) and the overall percent spore viability (% SV) are shown. (B) Bar graph showing spore viability in wild-type (gray) and *pch2Δ* (black) mutants containing the indicated *spo11* mutations. The *SPO11/SPO11*, *spo11-HA/spo11-HA*, *spo11-HA/spo11yf-HA*, and *spo11da-HA/spo11da-HA* alleles confer 100, 80, 30 and 20% total DSB levels, respectively, in the *PCH2* background [32].

We analyzed the *pch2Δ* phenotype in two different strain backgrounds at 30°C. In the EAY1108/1112 (EAY) SK1 congenic strain background, one large chromosome (XV, 1095 kb) is marked, whereas large (VII, 1040 kb), medium (VIII, 582 kb), and small (III, 333 kb) chromosomes are marked in the SK1 isogenic NHY942/943 (NHY) strain background ([10],[11]; Figure 1A, Figure 2A; Table S1). Similar to previous studies, *pch2Δ* mutants show wild-type spore viability (∼95%; [26],[47],[51]; Figure 3, Figure S1).

**Table 1:** Genetic map distances calculated from four-spore viable tetrads.

	4-spore viable tetrads	PD	TT	NPD	cM	SE	to wild-type	p values to *pch2Δ*	to *msh5Δ*
**Chromosome XV**									
***URA3-LEU2***									
wild-type	1087	607	456	5	22.8	1.0			
*pch2Δ*	1015	563	423	18	26.4	1.4	<0.0001		
*msh5Δ*	757	643	76	1	5.7	0.7	<0.0001	<0.0001	
*pch2Δ msh5Δ*	94	79	14	0	7.5	1.9	<0.0001	<0.0001	0.3620
***LEU2-LYS2***									
wild-type	1087	496	569	3	27.5	0.9			
*pch2Δ*	1015	395	561	39	40.0	1.8	<0.0001		
*msh5Δ*	757	562	155	3	12.0	1.0	<0.0001	<0.0001	
*pch2Δ msh5Δ*	94	74	17	1	12.5	3.7	<0.0001	<0.0001	0.5576
***LYS2-ADE2***									
wild-type	1087	803	263	2	12.9	0.8			
*pch2Δ*	1015	649	344	7	19.3	1.1	<0.0001		
*msh5Δ*	757	659	61	0	4.2	0.5	<0.0001	<0.0001	
*pch2Δ msh5Δ*	94	82	10	0	5.4	1.6	<0.0001	<0.0001	0.7296
***ADE2-HIS3***									
wild-type	1087	343	709	16	37.7	1.2			
*pch2Δ*	1015	243	638	115	66.7	2.3	<0.0001		
*msh5Δ*	757	496	215	9	18.7	1.5	<0.0001	<0.0001	
*pch2Δ msh5Δ*	94	54	37	2	26.3	4.9	<0.0001	<0.0001	0.0845
**Chromosome III**									
***HIS4-LEU2***									
wild-type	572	414	142	2	13.8	1.2			
*pch2Δ*	611	427	150	3	14.5	1.3	0.8093	0.0001	
*spo11-HA*	518	411	96	1	10.0	1.0	0.0016		
*pch2Δ spo11-HA*	556	438	100	1	9.8	1.0	0.0005	0.9772	0.0001
***LEU2-CEN3***									
wild-type	572	499	70	0	6.2	0.7			
*pch2Δ*	611	503	100	0	8.3	0.8	0.0092	0.9995	
*spo11-HA*	518	429	85	0	8.3	0.8	0.0200		
*pch2Δ spo11-HA*	556	453	96	2	9.8	1.1	0.0004	0.1311	0.0939
***CEN3-MAT***									
wild-type	572	405	164	1	14.9	1.1			
*pch2Δ*	611	440	162	1	13.9	1.0	0.5796	0.0007	
*spo11-HA*	518	398	112	5	13.8	1.5	<0.0001		
*pch2Δ spo11-HA*	556	487	68	0	6.1	0.7	<0.0001	<0.0001	<0.0001
**Chromosome VII**									
***TRP5-CYH2***									
wild-type	572	202	352	12	37.5	1.9			
*pch2Δ*	611	153	371	67	65.4	3.6	<0.0001	<0.0001	
*spo11-HA*	518	155	336	22	45.6	2.6	0.0008		
*pch2Δ spo11-HA*	556	173	335	43	53.8	3.2	<0.0001	0.0006	0.0014
***CYH2-MET13***									
wild-type	572	452	104	0	9.4	0.8			
*pch2Δ*	611	372	156	5	17.5	1.5	<0.0001	<0.0001	
*spo11-HA*	518	378	102	0	10.6	0.9	0.3720		
*pch2Δ spo11-HA*	556	433	82	1	8.5	1.0	0.0730	0.0036	<0.0001
***MET13-LYS5***									
wild-type	572	335	209	5	21.8	1.5			
*pch2Δ*	611	273	243	17	32.4	2.4	<0.0001	<0.0001	
*spo11-HA*	518	277	204	1	21.8	1.3	0.0295		
*pch2Δ spo11-HA*	556	343	161	11	22.0	2.1	0.0005	<0.0001	<0.0001
**Chromosome VIII**									
***CEN8-THR1***									
wild-type	572	319	221	2	21.5	1.3			
*pch2Δ*	611	302	228	7	25.1	1.7	0.0133	0.0227	
*spo11-HA*	518	309	195	3	21.0	1.4	0.4436		
*pch2Δ spo11-HA*	556	375	161	4	17.1	1.4	<0.0001	0.0001	<0.0001
***THR1-CUP1***									
wild-type	572	278	260	1	24.7	1.2			
*pch2Δ*	611	189	305	31	46.8	3.0	<0.0001	<0.0001	
*spo11-HA*	518	186	312	7	35.1	1.8	<0.0001		
*pch2Δ spo11-HA*	556	227	292	20	38.2	2.5	<0.0001	<0.0001	0.0027

The map distances in cM between the indicated markers and the number of each tetrad type observed (as calculated by RANA software; Argueso *et al.* [11]) are shown. Chromosome XV data were obtained from EAY background strains; chromosome III, VII and VIII data were obtained from NHY background strains. The Stahl lab online tools (http://www.molbio.uoregon.edu/~fstahl/) were used to calculate the genetic distances and standard errors (SE). p values for G-tests comparing the tetrad type distributions for all mutant combinations were calculated using the spreadsheet available from The Online Handbook of Biological Statistics (http://udel.edu/~mcdonald/statintro.html).

In the EAY strain background, the total map distance across four intervals on chromosome XV was 152 cM in *pch2Δ* compared to 101 cM in wild-type (Figure 1; Table 1). Increased crossing over in *pch2Δ* was statistically significant in all four intervals (G-test where p<0.017 is considered significant due to Dunn-Sidak correction for multiple tests; see Table 1 for p values). Similar results were observed on the large (VII) and medium (VIII) chromosomes in the NHY background (Figure 2B; Table 1). Significantly more crossing over was observed in each of three intervals on chromosome VII, raising the map distance of the marked region from 69 cM in wild-type to 115 cM in *pch2Δ* (G-test where p<0.017 is considered significant due to Dunn-Sidak correction for multiple tests; see Table 1 for p values). For chromosome VIII, statistically significant increases in crossing over were observed in both genetic intervals, raising the map distance from 46 cM in wild-type to 72 cM in *pch2Δ*. The increases in crossing over observed in *pch2Δ* on chromosomes XV, VII, and VIII resulted from an increase in both tetratype and non-parental ditype tetrads (Table 1). These data argue against the increase being due to multiple COs resulting from a single initiating DSB [52].

The effect of *pch2Δ* on crossing over on the small chromosome III was similar to that reported by San Segundo and Roeder [26], who saw no effect of the *pch2Δ* mutation on crossing over. We observed a significant increase in crossing over in *pch2Δ* in only one (*LEU2-CEN3*) of three genetic intervals (Figure 2B; Table 1). However, the overall map distance for the marked region in *pch2Δ* was 37 cM, which was not significantly different from wild-type (35 cM).

#### Gene conversion is elevated in *pch2Δ* mutants

The *pch2Δ* mutation conferred an increase in gene conversion for 15 of the 17 markers that were examined (Table 2). Two markers with the most dramatic increases in gene conversion were *met13* (2.4% in wild-type, 11.0% in *pch2Δ*) and *thr1* (5.1% in wild-type, 11.9% in *pch2Δ*), both in the NHY strain background. Tetrads in which high levels of gene conversion were observed (*THR1*, chromosome VIII, *MET13*, chromosome VII) were analyzed for exchange of flanking markers (Table 3; see [32]). For example, tetrads containing *MET13* gene conversions were scored in the CO class if *LYS5* and *CYH2* markers were non-parental ditype or tetratype, but were in the NCO class if those markers were parental ditype. A ratio of CO:NCO was then computed from these classes. At *MET13*, the CO:NCO ratio was 1.8 in wild-type and 2.6 in *pch2Δ*, but this difference was not statistically significant (G-test where p<0.05 is significant). At *THR1* the ratio was 1.9 in wild-type and 9.4 in *pch2Δ* (p<0.0001; Table 3). Assuming no change in DSB formation in *pch2Δ* (see below), these data suggest that at least for the *THR1* locus, the increase in crossing over observed in *pch2Δ* was accompanied by a relative decrease in noncrossover events. However, more extensive genetic analyses at multiple loci, using markers that can eliminate incidental COs, will be required to solidify this observation (see Discussion).

**Table 2:** *pch2Δ* increases the frequency of aberrant marker segregation.

Chromosome XV	Tetrads	*TRP1*	*URA3*	*LEU2*	*LYS2*	*ADE2*	*HIS3*	Total
wild-type	1087	0.0	0.0	0.2	0.6	0.1	0.8	1.7
*pch2Δ*	1015	0.3	0.2	1.1	0.4	0.4	1.5	3.9
*msh5Δ*	757	0.1	0.1	1.6	1.2	0.8	1.2	5.0
*pch2Δ msh5Δ*	94	0.0	0.0	1.1	1.1	1.1	0.0	3.3

Chromosome III	Tetrads	*HIS4*	*LEU2*	*ADE2*	*MATa*	Total
wild-type	572	2.1	0.3	0.2	0.2	2.8
*pch2Δ*	611	3.8	1.3	0.0	1.3	6.4
*spo11-HA*	518	1.2	0.8	0.0	0.6	2.6
*pch2Δ spo11-HA*	556	2.2	0.9	0.0	0.2	3.3
**Chromosome VII**	**Tetrads**	***LYS5***	***MET13***	***CYH2***	***TRP5***	**Total**
wild-type	572	1.6	2.4	0.3	0.7	5.0
*pch2Δ*	611	1.8	11.0	1.8	1.5	16.1
*spo11-HA*	518	0.2	6.8	0.6	0.4	8.0
*pch2Δ spo11-HA*	556	0.4	7.0	0.2	0.7	8.3
**Chromosome VIII**	**Tetrads**	***URA3***	***THR1***	***CUP1***	**Total**	
wild-type	572	0.2	5.1	0.7	6.0	
*pch2Δ*	611	0.2	11.9	2.1	14.2	
*spo11-HA*	518	0.0	2.1	0.4	2.5	
*pch2Δ spo11-HA*	556	0.0	2.9	0.2	3.1	

The percent of non 2:2 marker segregations were calculated for the indicated loci. Chromosome XV data were obtained from EAY background strains; chromosomes III, VII, and VIII data were obtained from NHY background strains. Most events were 3:1 or 1:3 gene conversions although one 4:0 event in the EAY background and two 4:0 events in the NHY background were observed in *pch2Δ* mutants. In addition, one post-meiotic segregation event (5:3) was observed in the *pch2Δ spo11-HA* mutant.

**Table 3:** CO:NCO ratio of markers flanking gene conversion events on chromosomes VII and VIII.

	4-spore viable tetrads	*MET13* conversions	CO (*LYS5-CYH2*)	NCO (*LYS5-CYH2*)	CO:NCO ratio	p value
wild-type	572	14	9	5	1.8	
*pch2Δ*	611	65	47	18	2.6	0.169
	**4-spore viable tetrads**	***THR1*** ** conversions**	**CO (** ***CEN8-CUP1*** **)**	**NCO (** ***CEN8-CUP1*** **)**	**CO:NCO ratio**	**p value**
wild-type	572	29	19	10	1.9	
*pch2Δ*	611	73	66	7	9.4	<0.0001

Tetrads containing a gene conversion at the *MET13* or *THR1 loci* were analyzed in wild-type and *pch2Δ* mutants in the NHY background. The markers flanking the gene conversion event were scored as CO (TT or NPD) or NCO (PD) and the CO:NCO ratio was calculated at each site. The wild-type and *pch2Δ* CO and NCO numbers were compared using a G-test and the p values are shown.

### Genetic analysis of meiotic CO control

#### Pch2 is required for wild-type levels of CO interference

The crossover phenotype of *pch2Δ* mutants, increased crossing over on large chromosomes, encouraged us to test a role for Pch2 in CO interference. As shown below, our data, and work by Joshi *et al*. [53], indicate that *pch2Δ* mutants are defective in CO interference. We employed three methods to measure CO interference. First, we measured the NPD ratio for those loci in which a significant number of NPD events were expected (>10; chromosome III data are thus excluded) using Stahl's “Better Way” calculator. This method compares the observed number of each tetrad class (NPD, PD and TT), to the numbers expected if CO distribution was random [54]–[56]. In the absence of CO interference, the NPD ratio is expected to be one. Values significantly less than one reflect the presence of CO interference with smaller numbers indicating stronger interference. We found *pch2Δ* mutants had a larger NPD ratio than wild-type in all genetic intervals in both strain backgrounds (Table 4). In the EAY strain, statistically significant levels of interference were seen in all genetic intervals in wild-type and in two of three intervals in *pch2Δ* (where p<0.05 is considered significant; see Table 4). In the NHY strain interference was seen in all four intervals in wild-type, but in only one interval in *pch2Δ* (Table 4). These results are similar to those reported for a previously identified interference mutant *tid1*. Shinohara and colleagues found that *tid1* mutants showed larger NPD ratios than wild-type in all six genetic intervals assayed and a decrease in the number of intervals where interference was statistically significant, from five in wild-type to three in *tid1* [34],[57].

**Table 4:** Interference calculations using NPD ratios.

	4-spore viable tetrads	NPD obs.	NPD exp.	obs./exp.	p	I
**Chromosome XV**						
***URA3-LEU2***						
wild-type	1087	5	27.7	0.18	<0.0001	YES
*pch2Δ*	1015	18	28.7	0.63	0.0212	YES
***LEU2-LYS2***						
wild-type	1087	3	43.2	0.07	<0.0001	YES
*pch2Δ*	1015	39	58.8	0.66	0.0011	YES
***ADE2-HIS3***						
wild-type	1087	16	74.8	0.21	<0.0001	YES
*pch2Δ*	1015	115	117.0	0.98	0.7522	NO
**Chromosome VII**						
***TRP5-CYH2***						
wild-type	572	12	36.0	0.33	<0.0001	YES
*pch2Δ*	611	67	66.4	1.00	0.9191	NO
*spo11-HA*	518	22	41.6	0.53	<0.0001	YES
*pch2Δ spo11-HA*	556	43	47.9	0.90	0.3362	NO
***MET13-LYS5***						
wild-type	572	5	11.8	0.42	0.0256	YES
*pch2Δ*	611	17	20.0	0.85	0.4298	NO
*spo11-HA*	518	1	12.0	0.08	0.0003	YES
*pch2Δ spo11-HA*	556	11	8.7	1.26	0.3834	NO
**Chromosome VIII**						
***CEN8-THR1***						
Wild-type	572	2	12.6	0.16	0.0007	YES
*pch2Δ*	611	7	14.9	0.47	0.0185	YES
*spo11-HA*	518	3	10.7	0.28	0.0077	YES
*pch2Δ spo11-HA*	556	4	7.0	0.57	0.2138	NO
***THR1-CUP1***						
wild-type	572	1	17.5	0.06	<0.0001	YES
*pch2Δ*	611	31	37.4	0.83	0.1725	NO
*spo11-HA*	518	7	30.2	0.23	<0.0001	YES
*pch2Δ spo11-HA*	556	20	29.1	0.69	0.0359	YES

The number of NPDs was compared to the expected number using the Stahl Online Laboratory “Better Way” calculator (http://www.molbio.uoregon.edu/~fstahl/). Chromosome XV data were obtained from EAY background strains; chromosomes III, VII, and VIII data were obtained from NHY background strains. The total number of 4-spore viable tetrads used for analysis is shown. The number of PD, NPD and TT tetrads can be found in Table 1. p values were calculated from the chi-square values provided by the “Better Way” program using Vasserstats (http://faculty.vassar.edu/lowry/VassarStats.html) chi-square to p calculator with one degree of freedom. “I” indicates if interference was statistically detectable.

Second, we measured the coefficient of coincidence (COC). This method compares the observed number of times that a CO occurs in each of two adjacent genetic intervals to the number of such double COs expected due to chance. In the absence of interference, the COC value is expected to equal one. Values significantly less than one indicate interference, with smaller numbers indicating stronger interference. All intervals in the two strain backgrounds displayed COC values that were higher in *pch2Δ* than in wild-type (Table 5). For three intervals, interference could not be detected in either wild-type or *pch2Δ*. In one interval, interference was seen in wild-type, but not *pch2Δ*. For the remaining four intervals, interference was seen in both wild-type and *pch2Δ*, but was weaker in *pch2Δ*.

**Table 5:** Interference calculations using coefficients of coincidence.

	4-spore viable tetrads	DCO obs.	DCO exp.	COC	p	I
**Chromosome XV**						
***URA3-LEU2-LYS2***						
wild-type	1087	177	246.9	0.72	<0.0001	YES
*pch2Δ*	1015	232	265.9	0.87	0.017	YES
***LEU2-LYS2-ADE2***						
wild-type	1087	65	141.9	0.46	<0.0001	YES
*pch2Δ*	1015	181	210.6	0.86	0.024	YES
***LYS2-ADE2-HIS3***						
wild-type	1087	158	179.9	0.88	0.080	NO
*pch2Δ*	1015	258	265.4	0.97	0.624	NO
**Chromosome III**						
***HIS3-LEU2-CEN3***						
wild-type	572	5	17.7	0.28	0.003	YES
*pch2Δ*	611	14	25.4	0.55	0.027	YES
*spo11-HA*	518	8	16.0	0.50	0.057	NO
*pch2Δ spo11-HA*	556	11	18.0	0.61	0.119	NO
***LEU2-CEN3-MAT***						
wild-type	572	17	20.3	0.84	0.529	NO
*pch2Δ*	611	31	27.0	1.15	0.490	NO
*spo11-HA*	518	16	19.3	0.83	0.516	NO
*pch2Δ spo11-HA*	556	17	12.0	1.42	0.190	NO
**Chromosome VII**						
***TRP5-CYH2-MET13***						
wild-type	572	59	68.1	0.87	0.267	NO
*pch2Δ*	611	122	132.3	0.92	0.337	NO
*spo11-HA*	518	63	76.1	0.83	0.119	NO
*pch2Δ spo11-HA*	556	55	60.8	0.91	0.472	NO
***CYH2-MET13-LYS5***						
wild-type	572	20	40.5	0.49	0.001	YES
*pch2Δ*	611	69	78.5	0.88	0.276	NO
*spo11-HA*	518	17	43.4	0.39	<0.0001	YES
*pch2Δ spo11-HA*	556	25	27.7	0.91	0.667	NO
**Chromosome VIII**						
***CEN8-THR1-CUP1***						
wild-type	572	67	108.0	0.62	<0.0001	YES
*pch2Δ*	611	125	150.4	0.83	0.019	YES
*spo11-HA*	518	85	125.1	0.68	<0.0001	YES
*pch2Δ spo11-HA*	556	76	95.5	0.80	0.032	YES

Chromosome XV data were obtained from EAY background strains; chromosomes III, VII and VIII data were obtained from NHY background strains. The number of double crossovers observed was compared to the expected number (as calculated by RANA software; [11]) for the EAY (A) and NHY (B) strain backgrounds. Two-tailed p values were calculated using the Vasserstats binomial properties calculator using a normal distribution. “I” indicates if interference was statistically detectable.

**Figure 4:**
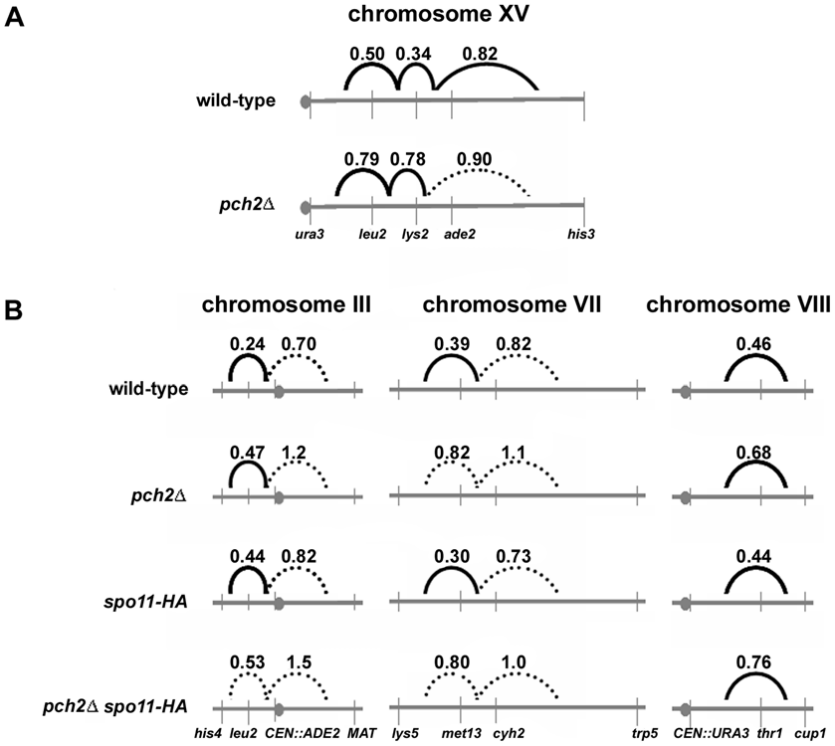
CO interference is reduced in absence of Pch2. Interference was measured using the method of Malkova *et al.* [32],[37] for strains in the EAY (A) and NHY (B) strain backgrounds. For each interval, the map distance was separately calculated for tetrads in which the adjacent interval had (+) or did not have (−) a CO event. If the two map distances are significantly different (G-test), then CO interference is present between the two intervals. The ratio of the CO+ to CO− map distances gives the strength of the interference, with values nearer to zero indicating stronger interference. The average interference ratio between adjacent genetic intervals is shown above the intervals. Solid lines indicate interference was statistically significant when either interval was used as the reference. The broken lines indicate interference was not statistically significant when one or both intervals were used as the reference. See Table S2 for raw data and statistical analyses.

Lastly, we employed the method of Malkova *et al.* [37] to analyze CO interference. This method compares the map distance calculated for a given interval when a CO has occurred in the adjacent interval to the map distance calculated for the same given interval when a CO has not occurred in the adjacent interval. In the absence of interference, these map distances are expected to be the same and a ratio of the map distances is equal to one. However, in the presence of interference, a CO in one interval would make a nearby CO less likely. This would cause the map distance ratio to be less than one, with smaller ratios resulting from stronger interference [37]. In both strain backgrounds the map distance ratios were larger in *pch2Δ* than wild-type for all adjacent interval pairs, indicating that, as seen with the NPD ratio and COC tests, *pch2Δ* disrupted CO interference (Figure 4; Table S2). In the EAY strain background, G-tests indicated that interference was statistically detectable in wild-type between all three interval pairs, but was detectable between only two interval pairs in *pch2Δ* (Figure 4A; Table S2). In the NHY strain background, interference was statistically detectable in *pch2Δ* for two out of five interval pairs, although it was weaker than in wild-type. For one interval pair, interference was not detected in *pch2Δ*, whereas it was present in wild-type. For the remaining two intervals, interference was not detected in wild-type or *pch2Δ* (Figure 4B; Table S2).

We saw no evidence of chromatid interference in any strain analyzed in this study. We also found no evidence for negative interference between genetic intervals on different chromosomes or between widely spaced intervals on the same chromosome in *pch2Δ,* suggesting that the decreases in positive interference we observed did not result from variability in recombination between meioses (data not shown; [58],[59]).

#### Pch2 is required for maintaining spore viability in *spo11* hypomorphs

Previously, Martini *et al.* [32] observed that CO levels were maintained at the expense of NCOs when meiotic DSBs became limiting in *spo11* hypomorphs showing reduced DSB levels (20–80%; [24],[32],[33],[60]). This homeostasis mechanism is thought to ensure obligate CO formation between all homologous chromosome pairs and thereby promote spore viability. If interference and homeostasis result from a common mechanism, a mutation disrupting CO interference (e.g. *pch2Δ*) would severely compromise the spore viability of *spo11* hypomorph strains [33].

We tested the effect of the *pch2Δ* mutation on the spore viability of *spo11* hypomorph strains (NHY background). As shown in Figure 3, spore viability was similar in wild-type (91%) and the *spo11-HA/spo11-HA* hypomorph (91%), which displays 80% of the wild-type level of DSBs. These results confirm work by Martini *et al.* [32]. Interestingly, the *pch2Δ/pch2Δ spo11-HA/spo11-HA* mutant displayed significantly lower spore viability, 73%, despite having CO levels (165 cM total) that were similar to *spo11-HA/spo11-HA* (166 cM) and above wild-type levels (150 cM; Figure 2 and Figure 3). Spore viability in *pch2Δ* strains was compromised even further, relative to *PCH2*, in strains bearing more defective *spo11* alleles (Figure 3; 86% spore viability in *spo11-HA/spo11yf-HA* vs. 16% in *pch2Δ/pch2Δ spo11-HA/spo11yf-HA*; 50% in *spo11da-HA/spo11da-HA* vs. 1% in *pch2Δ/pch2Δ spo11da-HA/spo11da-HA*). We also observed that the *pch2-G319A* mutation, which maps to the Walker A motif and is predicted to disrupt Pch2 ATP binding/hydrolysis activities [26],[47], is unable to complement the *pch2Δ* mutation (S. Zanders, J. Olszewski, M. Dowicki, E. Alani, unpublished data).

The excess of tetrads with 4, 2, and 0 viable spores per tetrad observed in the *pch2Δ spo11*-hypomorph double mutants suggests that the spore death results from MI chromosome nondisjunction, although we are unable to rule out additional causes (see Discussion). In support of this, we observed that 68% (n = 130) of two-spore viable tetrads were sisters in the *pch2Δ/pch2Δ spo11-HA/spo11-HA* double mutant, as determined by the centromere-linked markers *URA3* and *ADE2*. This was higher than what we observed in *spo11-HA/spo11-HA* and *pch2Δ/pch2Δ* where only 35% (n = 52) and 48% (n = 29), respectively, of the two-spore viable tetrads were sisters (G-test where p<0.025 is significant due to correction for multiple comparisons). We also observed significantly more (9/936) tetrads in which chromosome III had undergone MI nondisjunction, as determined by the *ADE2* centromere-linked marker and an inability to mate, in the *pch2Δ/pch2Δ spo11-HA/spo11-HA* double mutant as compared to *spo11-HA/spo11-HA* (0/649) and *pch2Δ/pch2Δ* (1/707). Together these observations are consistent with Pch2 regulating the distribution of CO events required to promote MI disjunction.

#### The interference defect in *pch2Δ* is not dependent upon extra COs

Previous studies suggested that the CO interference mechanism is intact in *ndj1* and *csm4* mutants but appears to be disrupted due to excess non-interfering COs [7],[8],[10],[15],[23],[45]. We entertained such a mechanism to explain the interference defect in *pch2Δ* by examining interference in *pch2Δ spo11* hypomorphs and *pch2Δ* mutants defective in the non-interfering (Mms4-Mus81) and interfering (Msh4-Msh5) CO pathways. As described below, our data do not support the excess non-interfering CO hypothesis.

First, *pch2Δ/pch2Δ spo11-HA/spo11-HA* mutants showed interference defects similar to *pch2Δ /pch2Δ* (Figure 2, Figure 4B; Table 4, Table 5, Table S2). This defect was seen even though the total number of COs decreased from 224 cM in *pch2Δ*/*pch2Δ* to 165 cM in *pch2Δ*/*pch2Δ spo11-HA/spo11-HA*.

Second, we tested if the decreased interference in *pch2Δ* was due to additional COs formed through the Mms4-Mus81 non-interfering CO pathway. This was done by analyzing *pch2Δ mms4Δ* and *pch2Δ msh5Δ* tetrads in the EAY strain background. The *pch2Δ mms4Δ* mutant had considerably lower spore viability (18%) than the *mms4Δ* mutant (53%; Figure S1). Overall, the recombination frequency of *pch2Δ mms4Δ* spores was about 14% higher than *mms4Δ* spores, but still lower than *pch2Δ* (Figure 1C; Table S3). In three out of four genetic intervals, the recombination frequencies were significantly higher in *pch2Δ mms4Δ* than in *mms4Δ* spores (G-test where p<0.025 is considered significant due to Dunn-Sidak correction for multiple comparisons; Table S3). These data suggest that the elevated crossing over seen in *pch2Δ* was not solely due to Mms4-Mus81-specific crossing over.

The spore viability of the *pch2Δ msh5Δ* mutant was 26%, compared to 36% for the *msh5Δ* single mutant (Figure S1). Like the *mms4Δ* mutant, overall CO frequencies (in tetrads and spores) were higher in the *pch2Δ msh5Δ* double mutant than in *msh5Δ* (∼30%; Figure 1B and 1C; Table 1, Table S3), but were much lower than in *pch2Δ*. When only data from complete tetrads were compared, there were no statistically significant difference between *msh5Δ* and *pch2Δ msh5Δ* (G-test where p<0.025 is considered significant due to Dunn-Sidak correction for multiple tests). However, when data from all surviving spores were analyzed, *pch2Δ msh5Δ* had significantly higher recombination frequencies than *msh5Δ* in two out of the four genetic intervals (G-test where p<0.025 is considered significant due to Dunn-Sidak correction for multiple tests). A caveat to these analyses is that the low spore viabilities observed in both the *pch2Δ mms4Δ* and *pch2Δ msh5Δ* mutants constrained analysis to a selected minority of meiotic products. Together, these data are consistent with COs in *pch2Δ* requiring both Mms4-Mus81 and Msh4-Msh5 pathways and argue against the idea that *pch2Δ* mutants show decreased CO interference due to additional COs formed through a non-interfering CO pathway.

### *pch2Δ* does not increase DSB formation at two sites

Previous work indicated that *pch2Δ* mutants show delays in meiotic DSB repair; thus, a time course comparison of DSB levels in meiotic prophase between *pch2Δ* and wild-type could be misleading [27],[47],[61]. Wu and Burgess [47] assayed DSB formation at the well-characterized *HIS4LEU2* hotspot in wild-type and *pch2Δ* in a *sae2Δ* strain background where DSBs are formed but not resected or repaired. They reported that wild-type and *pch2Δ* strains displayed similar DSB levels. More recently, the Hochwagen group, using microarray analysis, observed increases in DSB formation in *pch2Δ* surrounding the rDNA on chromosome XII, but nowhere else in the genome (A. Hochwagen personal communication).

We assayed DSB formation in *pch2Δ* mutants at the *YCR048W* hotspot on chromosome III and near the centromere on chromosome XV [42],[62],[63]. These experiments were performed in a *dmc1Δ* background where DSBs are formed at wild-type levels and resected (eventually hyperresected), but not repaired [29],[42],[64]. This approach allowed us to assay total DSB at loci other than *HIS4LEU2*, where DSBs are thought to occur at saturating levels, and avoid the use of the *sae2Δ* background where maximal DSB levels may not be reached [29],[32],[42],[65]. One concern with performing this analysis in the *dmc1Δ* background is that two reports [26],[66] indicated that the checkpoint arrest seen in *dmc1* mutants is bypassed in *pch2 dmc1* strains; however, a more recent report [61] indicated that it is not. Our *pch2Δ dmc1Δ* mutants displayed a meiotic arrest as measured by a failure to form spores (< 0.6% spore formation for *pch2Δ dmc1Δ* vs. ∼90% for wild-type at T = 24 hrs). However as shown below, we observed a significant bypass of the *dmc1* arrest in *pch2Δ spo11-HA dmc1Δ* strains.

**Figure 5:**
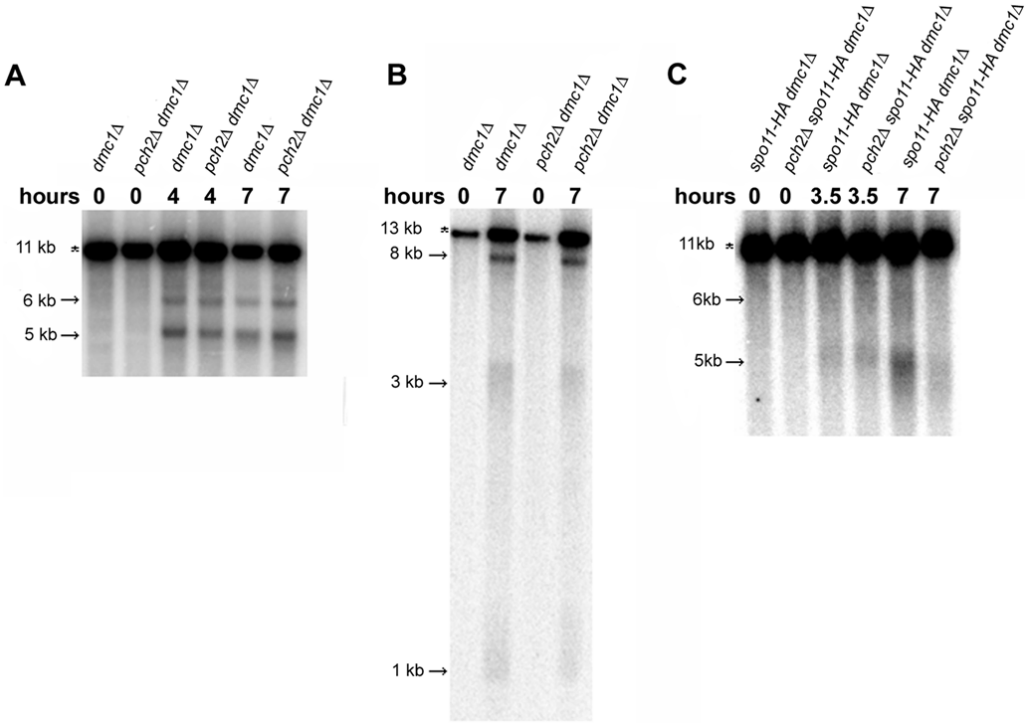
*pch2* does not appear to have increased levels of meiotic DSBs. Southern blots were performed to measure meiotic DSBs in *dmc1Δ*, *pch2Δ dmc1Δ, spo11-HA dmc1Δ*, and *pch2Δ spo11-HA dmc1Δ* strains. For the *YCR048w* hotspot on chromosome III (A) DNA was digested with *Bgl*II and probed with a chromosome III fragment (SGD coordinates 215,422-216,703, [63]). For the *CEN15* hotspot (B), DNA was digested with *Sph*I and *Nhe*I and probed with a chromosome XV fragment (SGD coordinates 331,713-332,402, [42]). The parental bands are marked with asterisks and arrows show bands that form due to DSB formation. Approximate sizes for all bands are shown [42],[63]. The lanes on the *CEN15* blot have been reordered for easy comparison of the two strains. (C) Analysis of DSBs at the *YCR048W* hotspot at T = 3.5 and 7 hrs in *spo11-HA dmc1Δ* (EAY2562/EAY2563) and *pch2Δ spo11-HA dmc1Δ* (EAY2564/EAY2565) strains. A representative blot is shown. In side-by-side experiments the DSB levels at T = 7 hrs in *pch2Δ spo11-HA dmc1Δ* ranged from 30-90% (30, 61, 72, 76, 80, 89, and 90%) of the levels observed in *spo11-HA dmc1Δ*.

Quantification of DSB levels in the *dmc1Δ* background is difficult due to the extensive resection of the breaks. We therefore analyzed five independent cultures of *dmc1Δ* and *pch2Δ dmc1Δ* strains. Similar to previous work ([47]; A. Hochwagen personal communication), we saw no difference in DSB levels (% of total DNA) between *dmc1Δ* and *pch2Δ dmc1Δ* strains at the *YCR048W* (5 and 6 kb DSB bands; 19±6% for *dmc1Δ,* 18±5% for *pch2Δ dmc1Δ*) and *CEN15* (8 kb DSB band; 4.7±1.2% for *dmc1Δ*, 4.5±1.0% for *pch2Δ dmc1Δ*) hotspots (Figure 5A and 5B; T = 7 hrs in meiosis). It is important to note that Hochwagen *et al.* [61] reported that *pch2Δ dmc1Δ* mutants do not resect DSB ends as rapidly as *dmc1Δ*; however, such a difference in resection rate could only result in an overestimation of the level of DSBs in *pch2Δ dmc1Δ.* These data, together with previous work, suggest that the *pch2* mutation does not disrupt DSB levels in a *SPO11* background.

As shown above, the *pch2Δ* mutation severely compromised the spore viability of *spo11* hypomorph strains. Because some *spo11* mutations confer semi-dominant and conditional phenotypes, as well as alter DSB patterns [60], we assayed DSB levels at *YCR048W* in *spo11-HA dmc1Δ* strains in the presence or absence of the *pch2Δ* mutation (Figure 5C). At T = 3.5 hrs in meiosis, similar DSB levels were observed in *pch2Δ spo11-HA dmc1Δ* (16%) and *spo11-HA dmc1Δ* (15%) strains. However, at T = 7 hrs, lower levels were observed in *pch2Δ spo11-HA dmc1Δ* (13±6 %; seven independent cultures) compared to *spo11-HA dmc1Δ* (18±6%; seven independent cultures). In time courses performed side by side, *pch2Δ spo11-HA dmc1Δ* strains displayed 30 to 90% of the *spo11-HA dmc1Δ* levels at T = 7 hrs. Such variability was not observed in side-by-side experiments involving *pch2Δ dmc1Δ* and *dmc1Δ* strains. As shown below and analyzed in the Discussion, we attribute the variability in DSB levels to the bypass of the *dmc1Δ* arrest in *pch2Δ spo11-HA dmc1Δ*. This was determined by measuring the completion of the MI division in *spo11-HA dmc1Δ* and *pch2Δ spo11-HA dmc1Δ* strains. At T = 28 hrs in meiosis, only 1-2% of *spo11-HA dmc1Δ* strains completed MI; this indicates that the *dmc1Δ* arrest is maintained in these strains. For *pch2Δ spo11-HA dmc1Δ*, at T = 4.5 hrs, no cells (n>200) had completed the MI division. However, at T = 6.5 hrs, 8 to 30% of the cells completed MI, and these values increased to 54 to 60% (with similar spore formation levels) at T = 28 hrs. As predicted for a *dmc1Δ* mutant, the spores produced by *pch2Δ spo11-HA dmc1Δ* were inviable.

### Analysis of meiotic progression

#### The *spo11-HA* hypomorph suppresses the MI delay of *pch2Δ*

**Figure 6:**
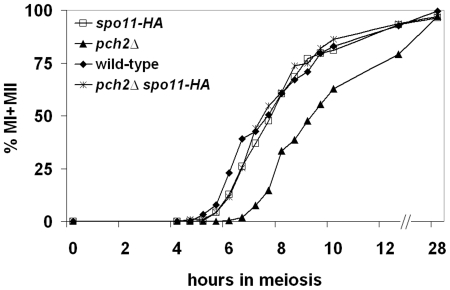
The *pch2* *Δ* MI delay is suppressed by the *spo11-HA* hypomorph. Synchronous cultures were induced to undergo meiosis and progression past MI (MI±MII) was assayed in the NHY background for wild-type (diamonds), *spo11-HA* (open squares), *pch2Δ* (triangles), and *pch2Δ spo11-HA* (stars; Materials and Methods). A representative time course is shown.

Wu and Burgess [47] showed that the *pch2Δ* MI delay is suppressed by a null mutation in the mitotic and meiotic checkpoint gene *RAD17*. The delay is also suppressed by the *spo11Δ* mutation [47],[67]. One interpretation of these and our data is that the greater than wild-type number of COs in *pch2Δ,* rather than a recombination-associated DNA aberration inherent to the mutant, triggers the Rad17-dependent checkpoint. If the additional time required to complete the additional COs causes the delay in *pch2Δ*, then reducing the number of recombination events by lowering the number of DSBs should suppress the delay. We assayed MI division timing in *pch2Δ*/*pch2Δ spo11-HA/spo11-HA* mutants displaying total CO levels (165 cM) that are somewhat similar to wild-type (150 cM) but significantly lower than *pch2Δ*/*pch2Δ* (224 cM; Figure 2B; [24],[32]). *pch2Δ*/*pch2Δ spo11-HA/spo11-HA* strains progressed through meiosis with timing indistinguishable from *spo11-HA/spo11-HA* and wild-type (Figure 6). These data suggest there are no inherent recombination defects recognized by a Rad17-dependent checkpoint in *pch2Δ* mutants, unless the defect appears only when DSBs are at wild-type levels [32, but see 27]. We favor the idea that the MI delay in *pch2Δ* is caused by the prolonged recombination period needed to generate the additional COs observed in *pch2Δ*. Alternatively, the extra COs observed in *pch2Δ* could result from, rather than cause, the MI delay [68]. In this case, it is unclear what could be eliciting the delay in *pch2Δ*. Importantly, the fact that the *pch2Δ spo11-HA* double mutant has wild-type MI timing *and* disrupted CO interference (Figure 4, Figure 6; Table 4, Table 5, Table S2) demonstrates that the interference defects observed in *pch2Δ* are not simply the result of a prolonged CO designation period [68].

## Discussion

In this study we show that *pch2* mutants display elevated crossing over on medium and large chromosomes and are defective in CO interference. Based on this work, our initial studies suggesting an increased CO:NCO ratio in *pch2Δ* mutants (Table 3), and previous work [27],[32],[47], we hypothesize that the increase in COs in *pch2Δ* on the medium and large chromosomes results from a greater than normal proportion of DSBs being repaired as COs at the expense of non COs, due to the loss of CO interference, rather than an increase in initiating DSBs (Figure 5; Table 3). In other words, we propose Pch2-mediated CO control acts not only to uniformly space COs within the genome, but also to limit the overall number of COs. The same defect in *pch2Δ* that disrupts interference could lead to longer heteroduplex tracts, causing the increases in gene conversion frequencies observed in *pch2Δ.*.

**Figure 7:**
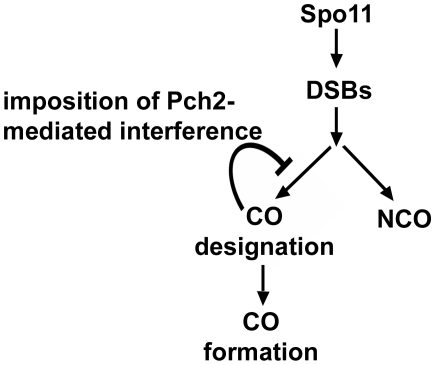
Model for interference-regulation of the CO vs. NCO decision. We propose that Pch2 acts to inhibit CO designation in a chromosomal region in response to a prior crossover-designated recombination event in that region. See text for details.

We favor a model in which Pch2 promotes wild-type levels of CO interference at the CO vs. NCO decision, which is though to occur in late leptotene, perhaps by acting in meiotic axis organization/assembly (Figure 7; [17],[18],[20]). In this model, CO designation at one site inhibits nearby DSBs from receiving CO designation; such a decision could then influence the Pch2-dependent domainal organization of Hop1 and Zip1 observed in pachytene [27; see below]. Two recent studies support the idea that Pch2 acts in early prophase. 1. Hochwagen *et al.* observed changes in DSB processing in *pch2* mutants [61]. 2. Shinohara and colleagues (personal communication) found that meiotic depletion of *CDC53* causes a defect in meiotic axis construction in leptotene, resulting in aberrant SC formation. A *pch2* mutation fully suppresses the SC construction defect of *CDC53* meiotic depletion, suggesting that Pch2 is a negative regulator of meiotic axis assembly.

Our proposal that Pch2 acts at the CO vs. NCO decision differs from interpretations presented by Börner *et al.* [27]. They examined NCO and CO formation at the *HIS4LEU2* hotspot on chromosome III in *pch2Δ* mutants using Southern blot analysis and found that CO levels were decreased and NCO levels were increased at this site [27],[69]. They also monitored progression through recombination at *HIS4LEU2* and found that *pch2Δ* mutants were delayed after SEI formation and accumulate SEIs and dHJs. Given these results and the finding that CO and NCO formation were coordinately delayed, Börner *et al.* [27] proposed that the meiotic delay in *pch2Δ* is caused by a defect downstream of the CO vs. NCO decision. We did not observe an effect of the *pch2Δ* mutation on crossing over on chromosome III. One explanation for this difference is that the Börner *et al.* [27] performed their analysis at *HIS4LEU2*, which was shown previously to lack CO homeostasis [32]. Additionally, the delay that they saw in processing recombination intermediates in *pch2Δ* may be due to an upstream defect at the CO vs. NCO decision. Specifically, the additional load of SEIs and dHJs that the recombination machinery must process in a *pch2Δ* mutant could delay their turnover genome-wide. It is important to note that we do not have a clear explanation for why CO levels on chromosome III are not elevated in *pch2Δ*. However, smaller chromosomes in yeast, such as chromosome III, have higher map distances per kb compared to larger chromosomes, and CO interference appears weaker on smaller chromosomes compared to larger ones [38],[39]. Thus, because interference is stronger on larger chromosomes, eliminating interference should have a more pronounced effect on CO levels on larger chromosomes, as was seen in our study.

### Meiotic axis organization appears to be conserved in *S. cerevisiae* and *C. elegans*

Martinez-Perez [70] recently reported a link between meiotic axis protein organization and CO interference in *C. elegans*. They analyzed the distribution patterns of the central element protein SYP-1 and the axial element proteins HTP-1 and HTP-2, which, like Hop1, are HORMA domain proteins. Analogous to observations made for Hop1 and Zip1 in yeast, Martinez-Perez *et al.* [70] found that the HTP axial element and the SYP-1 central element proteins sort into reciprocal domains on late pachytene chromosome axes. Based on the above, the finding that HTP1/2 is depleted at COs, the fact that Spo11 and Msh5 are required for domain formation, and the correlation seen between HTP1/2 depletion sites and chiasmata, Martinez-Perez *et al.* [70] suggest that HTP/SYP-1 domain boundaries mark CO sites. This information suggests that Hop1/Zip1 boundaries indicate where the CO/NCO decision marks subsequent CO sites. Such a model takes into account the finding that *C. elegans* displays only one domain of each type whereas *S. cerevisiae* contains a large number of alternating Zip1/Hop1 domains. This pattern is consistent with the fact that each chromosome pair in *C. elegans* typically enjoys a single CO whereas chromosome pairs in *S. cerevisiae* enjoy multiple COs (∼80–90 total COs in *S. cerevisiae* [30],[35] vs. six in *C. elegans* [21],[70]).

Based on observations presented in Martinez-Perez *et al*. [70] we suggest that the altered pattern of Hop1 and Zip1 localization on the chromosome axis seen in *pch2Δ* mutants results from, but is not the cause of, the increase in COs. In this interpretation, the defect in CO control in *pch2Δ* mutants leads to additional COs, reflected by a greater number of domains, thus making the axis distribution of Hop1 and Zip1 appear more uniform. This model fits with respect to the known timing of the CO vs. NCO decision [17],[18],[20],[21],[34],[35], and the finding that early Hop1 organization appears normal in *pch2Δ* mutants [27]. Testing such a model will require an examination of Hop1 and Zip1 localization patterns in strains (e.g. *pch2Δ spo11* hypomorphs) containing decreased levels of DSBs; our model predicts that the Hop1 and Zip1 domains would become more distinct due to fewer COs, although not completely like wild-type due to defects in CO interference.

### Why do *pch2* mutants show wild-type spore viability?

The wild-type spore viability seen in *pch2Δ* mutants suggests that Pch2-mediated CO control is not required to maintain the viability of yeast grown in lab conditions. We offer two explanations for this finding: 1) COs are present in excess (∼80–90 per cell) of the number needed for all homologs to receive an obligate CO (16 per cell). 2) The reduction in interference in *pch2Δ* is accompanied by, and likely causes, an increase in the overall number of COs. This increase in crossing over could compensate for distribution failures that jeopardized obligate CO formation ([30],[33]; Figure 3, Figure 4; Table 4, Table 5, Table S2). Our results and those of Martini *et al.* [32] demonstrate a buffered system in baker's yeast in which excess DSBs and COs lessen the need for interference to ensure obligate CO formation. Because of this buffer, obligate CO formation can be maintained if interference or DSBs are reduced, but not both (Figure 3; [32]). Such buffering may exist because the consequences of having too many COs are less severe than too few. For example, *pch2Δ* mutants have dramatic increases in CO levels, but show wild-type spore viability, whereas mutants that significantly decrease CO levels like *mlh3Δ*, have reduced spore viabilities due to MI nondisjunction [9],[11]. Future searches for mutants that disrupt the CO vs. NCO decision must be broadened to include genes with high spore viability or synthetic phenotypes with *spo11* hypomorphs.

Although the role of Pch2 in limiting CO levels, after the requisite number required for ensuring obligate CO formation is reached, is not required, it is likely to be advantageous. Too many COs, especially closely spaced ones, have been suggested to disrupt the sister chromatid cohesion required to create tension on the MI spindles and ensure proper homolog disjunction at MI [71],[72]. In addition, our data suggests that the CO limiting role of Pch2 also promotes timely meiotic progression, which could also be advantageous to cells (Figure 6).

What causes the loss in spore viability seen in *pch2Δ spo11* hypomorphs? *pch2Δ*/*pch2Δ spo11-HA/spo11-HA* strains displayed an excess of tetrads with 4, 2, and 0 viable spores, a high percentage of two-spore viable tetrads containing sisters, and an increased frequency of chromosome III nondisjunction. Our data are consistent with MI chromosome nondisjunction being a major component of the spore death phenotype, perhaps due to a failure to ensure obligate CO formation on all chromosomes. In such a model, when DSBs become limiting, the proper distribution of COs becomes even more critical to ensure obligate CO formation. Similar DSB levels were seen at *YCRO48W* at 3.5 hours in meiosis in *spo11-HA dmc1Δ* and *pch2Δ spo11-HA dmc1Δ*; however, by 7 hrs, fewer breaks were observed in the triple mutant (Figure 5C and 5D). Our DSB level measurements are not definitive due to the checkpoint bypass observed in the triple mutant. We provide two explanations for the triple mutant phenotype. In one scenario, early forming DSBs appear at wild-type levels while later-forming DSBs form at lower levels that are insufficient for sustained recombination checkpoint activation. In a second scenario, DSBs form normally, but undergo some level of Dmc1-independent, possibly intersister, repair that permits a bypass of the checkpoint. Such repair would not lead to MI disjunction-promoting chiasmata. Both of these scenarios are sufficient to explain the spore inviability seen in *pch2Δ spo11* hypomorphs (Figure 3). Future experiments to distinguish these hypotheses should include an analysis of meiotic Rad51foci in *spo11-HA dmc1Δ* and *pch2Δ spo11-HA dmc1Δ* strains [73].

We cannot rule out that other cellular defects contribute to the MI non-disjunction phenotype seen in *pch2Δ spo11* mutants. For example, both *pch2Δ* and *spo11* hypomorphs have SC defects, which could lead to CO control-independent synthetic phenotypes in the double mutants [24],[27]. It is also possible that Pch2 promotes MI disjunction by regulating sister chromatid cohesion establishment and/or removal, or by preventing/resolving chromosome entanglements [2]–[4],[68], or that some spore death in *pch2Δ spo11* hypomorphs is independent of MI non-disjunction.

### Additional factors are likely to act early in meiosis to establish CO interference

Interference mutants have been proposed to act downstream of the CO vs. NCO decision (e.g. *zip1, msh4*; Introduction; [22],[35]), or display an apparent defect in interference due to an increase in non-interfering COs (*ndj1, csm4*; [15],[35]). The only other yeast interference mutants that appear similar to *pch2Δ* are *tid1Δ* and *dmc1Δ*-*2µRad54* [34; but see 58]. We will focus on *tid1Δ*, because its CO phenotype is better characterized. Tid1/Rdh54 is a member of the Swi2/Snf2 family, and thus may act in meiotic chromatin axis remodeling, though this has yet to be tested [74]. *tid1Δ* mutants display moderate levels of spore viability (58% 4-spore viable tetrads), and Tid1 has been shown to be involved in the strand exchange step of recombination [57]. Similar to *pch2Δ*, *tid1Δ* mutants display a defect in interference and increased gene conversion. Also, like *pch2Δ*, CO levels in *tid1Δ* appear similar to wild-type on a small chromosome (III). On a medium-sized chromosome (V), *tid1Δ* mutants displayed wild-type CO levels in two intervals, but a significant (2.4-fold) increase in a third [34]. These data suggest that *tid1Δ* and *pch2Δ* have similar CO patterns. We are eager to test this hypothesis in the strain sets used in this study. Furthermore, we are intrigued by the idea that strand exchange and meiotic chromatin axis components are both required/involved in interference-regulation of the CO vs. NCO decision.

## Materials and Methods

### Media and yeast strains

Yeast strains are listed in Table S1. All strains were grown at 30°C on standard YPD (yeast peptone dextrose; [75]). The sporulation media was described previously [11],[68]. For tetrad genotyping, synthetic minimal selective media, synthetic complete media with 5 µM Cu, and YPD supplemented with complete amino acid mix and 3 mg/L cycloheximide were used [75]. When required, Geneticin (Invitrogen), nourseothricin (Hans-Knoll Institute fur Naturstoff-Forschung), and hygromycin B (Calbiochem) were added to YPD media as described [76],[77].

The EAY1108/EAY1112 SK1 congenic strain set is described in Argueso *et al.* [11], and the NHY942/NHY943 SK1 isogenic strain set is described in de los Santos *et al*. [10]. The *spo11* hypomorphic mutants were described by Diaz *et al*. [60] and Henderson and Keeney [24] although the NHY942/NHY943 strains containing these alleles, which are used in this work, are described in Martini *et al.* [32]. As in Martini *et al.* [32], we refer to *spo11-HA3His6* as *spo11-HA*, *spo11(D290A)-HA3His6* as *spo11da-HA*, and *spo11(Y135F)-HA3His6* as *spo11yf-HA*. Strains EAY2562-2565 are derivatives of a cross between EAY2260 and SKY633. The *msh5Δ*, *mms4Δ*, and *dmc1Δ* alleles used in this work were all complete open reading frame (ORF) deletions. The *pch2Δ* allele contains a deletion of amino acids 17–587 (in the 603 amino acid ORF). All deleted regions were replaced with *HPHMX4*, *KANMX4,* or *NATMX4* as shown in Table S1 [76],[77]. The deletion cassettes were made via PCR and integrated into the genome using standard techniques [78]. Details on strain construction and primer sequences are available on request.

### Tetrad analysis

Diploids for tetrad analysis were all made using the zero growth mating protocol [79]. The haploid parental strains were patched together on YPD for 4 hours and then spread on sporulation plates. The plates were incubated at 30°C for 2 days, after which tetrads were dissected. Tetrads from the EAY1108/EAY1112 strain background were dissected on synthetic complete media, whereas tetrads from the NHY942/NHY943 strain background were dissected on YPD media supplemented with complete amino acids. All tetrads were incubated 3–4 days at 30°C and then replica-plated to various selective media. The replica plates were scored after one day of incubation at 30°C. In the EAY strain background, the data for wild-type, *mms4Δ*, and *msh5Δ* were originally published in Argueso *et al.* [11]. In the NHY strain background, a subset of the wild-type data was originally published in Wanat *et al.* [68]. The distributions of each tetrad type were calculated using RANA software [11].

Genetic map distances±the standard error were calculated using the Stahl Laboratory Online Tools (http://www.molbio.uoregon.edu/~fstahl/) which utilizes the formula of Perkins [54]. The G-test spreadsheet, available from The Online Handbook of Biological Statistics (http://udel.edu/~mcdonald/statintro.html), was used to compare tetrad distribution patterns between strains. The Dunn-Sidak correction (p value of 0.05/ number of comparisons) was applied when multiple comparisons per data set were performed [80]. Recombination frequencies from spore data were calculated as described previously (RANA software; [11]), with p-values determined as above (http://udel.edu/~mcdonald/statintro.html).

Three different analyses were performed to measure interference. The NPD ratio (Table 4) was determined using the “Better Way” calculator (http://www.molbio.uoregon.edu/~fstahl/). This method compares the number of each tetrad type observed to the numbers expected if CO distribution was random and calculates a chi square value, which was converted to a p value using VassarStats (http://faculty.vassar.edu/lowry/VassarStats.html). Coefficients of coincidence (Table 5) were determined as described previously [11],[68]. Tetrads were sorted using Mactetrad 6.9 software to calculate interference via the Malkova *et al*. method ([37], Figure 4; Table S2).

### Meiotic time courses and DSB Southern blotting

For all time courses, a saturated YPD overnight culture from each strain to be analyzed was diluted in 200 ml YPA (2% potassium acetate) and grown for 17 hours. The YPA culture was then spun down, washed once in 1% potassium acetate and resuspended in 100 ml 1% potassium acetate (similar to [81]). All strains were grown in the same batches of media and treated identically. DAPI staining to analyze progression past MI (MI + MII) was performed as described [81]. Cells were visualized using an Olympus BX60 microscope and at least 200 cells were counted for each time point. DNA was isolated from meiotic cultures as described [29]. Southern blotting was performed using standard techniques [82]. The percent of DSB formation for four to six independent time courses (% of hybridizing bands±standard deviation, SD) was calculated using Image Quant software.

## Supporting Information

Figure S1 Spore viability distributions from tetrads in the EAY1108/EAY1112 strain background. The X-axes indicate the number of viable spores per tetrad and the Y-axes indicate the percent of tetrads represented by each class. The number of tetrads dissected (n) is indicated as well as the overall percentage of viable spores (SV). Strains homozygous for the indicated genotypes were analyzed (Table S1).
(5.82 MB TIF)

Table S1 The strains used are listed with their genotypes and the papers in which the strains were originally used. EAY1108 and EAY1112 and their derivatives are SK1 congenic strains. NHY942 and NHY943 and their derivatives are SK1 isogenic strains.
(0.07 MB DOC)

Table S2 Chromosome XV data were obtained from EAY background strains; chromosomes III, VII, and VIII data were obtained from NHY background strains. All pair-wise comparisons between adjacent intervals are shown. The top genetic interval listed in each box is the reference interval. All tetrads were divided into two classes: those with (CO+; i.e. NPD or TT) and those without (CO−; i.e. PD) an observable CO event within the reference interval using Mactetrad 6.9. The genetic size and standard error (SE) of the adjacent genetic interval (the lower listing at the top of the box) was then calculated for each class (CO+ and CO−) using the Stahl Laboratory Online Tools (http://molbio.uoregon.edu/~fstahl/). A ratio of the CO+/CO− class cM values was computed. Interference was considered significant if the CO+ and CO− classes were found to be significantly different via G-tests calculated using the spreadsheet available from The Online Handbook of Biological Statistics (http://udel.edu/~mcdonald/statintro.html).
(0.47 MB DOC)

Table S3 The recombination frequencies between the indicated markers and the number of parental and recombinant spores (as calculated by RANA software; Argueso et al. [11]) in the EAY strain background are shown. p values for G-tests comparing the recombinant and parental spore numbers for all mutant combinations were calculated using the spreadsheet available from The Online Handbook of Biological Statistics (http://udel.edu/~mcdonald/statintro.html).
(0.08 MB DOC)
