# Supplementary figures and images for: The pch2Δ Mutation in Baker's Yeast Alters Meiotic Crossover Levels and Confers a Defect in Crossover Interference
Source: PLoS Genet. 2009 Jul 24;5(7):e1000571. doi: 10.1371/journal.pgen.1000571 (PMC2709914; doi:10.1371/journal.pgen.1000571)

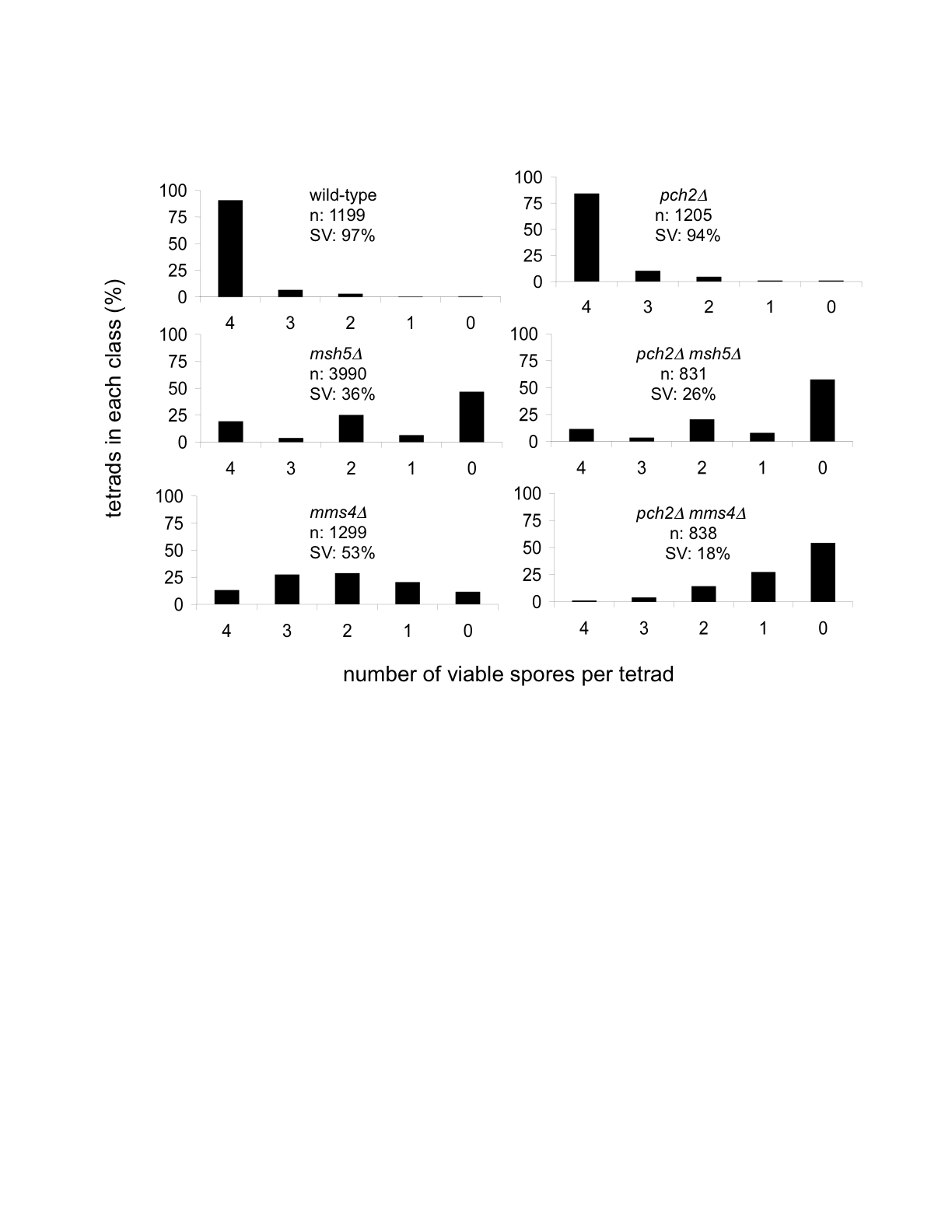

Supplement: Figure S1 — Spore viability distributions from tetrads in the EAY1108/EAY1112 strain background. The X-axes indicate the number of viable spores per tetrad and the Y-axes indicate the percent of tetrads represented by each class. The number of tetrads dissected (n) is indicated as well as the overall percentage of viable spores (SV). Strains homozygous for the indicated genotypes were analyzed (Table S1). (5.82 MB TIF) [file pgen.1000571.s001.tif]
