# Supplementary material for: The pch2Δ Mutation in Baker's Yeast Alters Meiotic Crossover Levels and Confers a Defect in Crossover Interference
Source: PLoS Genet. 2009 Jul 24;5(7):e1000571. doi: 10.1371/journal.pgen.1000571 (PMC2709914; doi:10.1371/journal.pgen.1000571)
Supplement: Table S1 — The strains used are listed with their genotypes and the papers in which the strains were originally used. EAY1108 and EAY1112 and their derivatives are SK1 congenic strains. NHY942 and NHY943 and their derivatives are SK1 isogenic strains. (0.07 MB DOC) [file pgen.1000571.s002.doc]

**Table S1. Yeast Strains.**

| **Strain name** | **Genotype** | **Source** |
| --- | --- | --- |
|  |  |  |
| EAY1108 | *MATa, ho::hisG, lys2, ura3, leu2::hisG, trp1::hisG, URA3-CEN15, iLEU2-chXV, iLYS2-chXV* | Argueso *et al.* [11] |
| EAY1975 | as EAY1108 except *pch2∆::NATMX4* | this work |
| EAY1281 | as EAY1108 except *msh5∆::NATMX4* | Argueso *et al.* [11] |
| EAY1167 | as EAY1108 except *mms4∆::KANMX4* | Argueso *et al.* [11] |
| EAY2332 | as EAY1281except *pch2∆::KANMX4* | this work |
| EAY2343 | as EAY1167 except *pch2∆::NATMX4* | this work |
|  |  |  |
| EAY1112 | *MATα, ho::hisG, lys2, ura3, leu2::hisG, trp1::hisG, ade2::hisG, his3::hisG, TRP1-CEN15* | Argueso *et al.* [11] |
| EAY1976 | as EAY1112 except *pch2∆::NATMX4* | this work |
| EAY1280 | as EAY1112 except *msh5∆::NATMX4* | Argueso *et al.* [11] |
| EAY1168 | as EAY1112 except *mms4∆::KANMX4* | Argueso *et al.* [11] |
| EAY2333 | as EAY1280 except *pch2∆::KANMX4* | this work |
| EAY2341 | as EAY1168 except *pch2∆::NATMX4* | this work |
|  |  |  |
| NH942 | *MATα, ho::hisG, ade2∆, can1, ura3(∆Sma-Pst), met13-B, trp5-S, CEN8::URA3, thr1-A, cup1^s^* | de los Santos *et al.* [10] |
| EAY2209 | as NH942 except *pch2∆::NATMX4* | this work |
| EAY2256 | as NH942 except *dmc1∆::KANMX4* | this work |
| EAY2260 | as EAY2209 except *dmc1∆::KANMX4* | this work |
| SKY633 | as NH942 except *MATa, spo11-HA3His6::KANMX4* | Martini *et al.* [32] |
| SKY1062 | as NH942 except *MATa, spo11(D290A)-HA3His6::KANMX4* | Martini *et al.* [32] |
| EAY2267 | as SKY633 except *pch2∆::NATMX4* | this work |
| EAY2269 | as SKY1062 except *pch2∆::NATMX4* | this work |
| EAY2545 | as EAY2267 except *dmc1*∆::*HPHMX4* | this work |
| EAY2546 | as SKY633 except *dmc1∆::HPHMX4* | this work |
| EAY2562 | as NH942 except *MATa*, *dmc1∆::KANMX4*, *spo11-HA3His6::KANMX4* | this work |
| EAY2563 | as NH942 except *dmc1∆::KANMX4, spo11-HA3His6::KANMX4* | this work |
| EAY2564 | as NH942 except *MATa*, *dmc1∆::KANMX4*, *spo11-HA3His6::KANMX4, pch2∆::NATMX4* | this work |
| EAY2565 | as NH942 except *dmc1∆::KANMX4, spo11-HA3His6::KANMX4, pch2∆::NATMX4* | this work |
|  |  |  |
| NH943 | *MATa, ho::hisG, ade2∆, ura3(∆Sma-Pst), leu2::hisG, CEN3::ADE2, lys5-P, cyh2^r^, his4-B* | de los Santos *et al.* [10] |
| EAY2210 | as NH943 except *pch2∆::NATMX4* | this work |
| EAY2257 | as NH943 except *dmc1∆::KANMX4* | this work |
| EAY2261 | as EAY2210 except *dmc1∆::KANMX4* | this work |
| SKY635 | as NH943 except *MATα, spo11-HA3His6::KANMX4* | Martini *et al.* [32] |
| SKY638 | as NH943 except *MATα, spo11(D290A)-HA3His6::KANMX4* | Martini *et al.* [32] |
| SKY665 | as NH943 except *MATα, spo11(Y135F)-HA3His6::KANMX4* | Martini *et al.* [32] |
| EAY2264 | as SKY635 except *pch2∆::NATMX4* | this work |
| EAY2265 | as SKY665 except *pch2∆::NATMX4* | this work |
| EAY2271 | as SKY638 except *pch2∆::NATMX4* | this work |
| EAY2535 | as SKY635 except *dmc1∆::HPHMX4* | this work |
| EAY2540 | as EAY2264 except *dmc1∆::HPHMX4* | this work |

**For Table S1:** The strains used are listed with their genotypes and the papers in which the strains were originally used. EAY1108 and EAY1112 and their derivatives are SK1 congenic strains. NHY942 and NHY943 and their derivatives are SK1 isogenic strains.
