# Supplementary material for: The pch2Δ Mutation in Baker's Yeast Alters Meiotic Crossover Levels and Confers a Defect in Crossover Interference
Source: PLoS Genet. 2009 Jul 24;5(7):e1000571. doi: 10.1371/journal.pgen.1000571 (PMC2709914; doi:10.1371/journal.pgen.1000571)
Supplement: Table S2 — Chromosome XV data were obtained from EAY background strains; chromosomes III, VII, and VIII data were obtained from NHY background strains. All pair-wise comparisons between adjacent intervals are shown. The top genetic interval listed in each box is the reference interval. All tetrads were divided into two classes: those with (CO+; i.e. NPD or TT) and those without (CO−; i.e. PD) an observable CO event within the reference interval using Mactetrad 6.9. The genetic size and standard error (SE) of the adjacent genetic interval (the lower listing at the top of the box) was then calculated for each class (CO+ and CO−) using the Stahl Laboratory Online Tools (http://molbio.uoregon.edu/~fstahl/). A ratio of the CO+/CO− class cM values was computed. Interference was considered significant if the CO+ and CO− classes were found to be significantly different via G-tests calculated using the spreadsheet available from The Online Handbook of Biological Statistics (http://udel.edu/~mcdonald/statintro.html). (0.47 MB DOC) [file pgen.1000571.s003.doc]

**Table S2. Interference calculations using the method of Malkova *et al.* [37].**

|  |  |  |  |  |  |  |  |  | **Chromosome XV** | | | |  | | |  |  |  |  |
| --- | --- | --- | --- | --- | --- | --- | --- | --- | --- | --- | --- | --- | --- | --- | --- | --- | --- | --- | --- |
|  |  |  |  |  |  |  |  |  |  |  |  |  | |  |  |  |  |  |  |
| **Reference** |  |  | *URA3-LEU2* |  |  |  |  |  |  |  |  | *URA3-LEU2* | |  |  |  |  |  |  |
|  |  |  | *LEU2-LYS2* |  |  |  |  |  |  |  |  | *LYS2-ADE2* | |  |  |  |  |  |  |
|  |  | PD | TT | NPD | cM |  | SE | ratio | p value |  | PD | TT | | NPD | cM |  | SE | ratio | p value |
|  | CO |  |  |  |  |  |  |  |  |  |  |  | |  |  |  |  |  |  |
| wild-type | + | 284 | 177 | 0 | 19.2 | ± | 1.1 |  |  |  | 346 | 115 | | 0 | 12.5 | ± | 1.0 |  |  |
|  | - | 212 | 392 | 3 | 33.8 | ± | 1.2 | 0.568 | <0.0001 |  | 457 | 148 | | 2 | 13.2 | ± | 1.1 | 0.947 | 0.212 |
|  |  |  |  |  |  |  |  |  |  |  |  |  | |  |  |  |  |  |  |
| *pch2*∆ | + | 203 | 214 | 18 | 37.0 | ± | 2.9 |  |  |  | 288 | 143 | | 4 | 19.2 | ± | 1.7 |  |  |
|  | - | 190 | 347 | 21 | 42.4 | ± | 2.4 | 0.873 | <0.0001 |  | 353 | 199 | | 3 | 19.6 | ± | 1.3 | 0.980 | 0.284 |
|  |  |  |  |  |  |  |  |  |  |  |  |  | |  |  |  |  |  |  |
| **Reference** |  |  | *URA3-LEU2* |  |  |  |  |  |  |  |  | *LEU2-LYS2* | |  |  |  |  |  |  |
|  |  |  | *ADE2-HIS3* |  |  |  |  |  |  |  |  | *URA3-LEU2* | |  |  |  |  |  |  |
|  |  | PD | TT | NPD | cM |  | SE | ratio | p value |  | PD | TT | | NPD | cM |  | SE | ratio | p value |
|  | CO |  |  |  |  |  |  |  |  |  |  |  | |  |  |  |  |  |  |
| wild-type | + | 163 | 294 | 4 | 34.5 | ± | 1.8 |  |  |  | 395 | 176 | | 1 | 15.9 | ± | 1.1 |  |  |
|  | - | 180 | 415 | 12 | 40.1 | ± | 1.6 | 0.860 | 0.007 |  | 212 | 280 | | 4 | 30.6 | ± | 1.6 | 0.520 | <0.0001 |
|  |  |  |  |  |  |  |  |  |  |  |  |  | |  |  |  |  |  |  |
| *pch2*∆ | + | 114 | 270 | 50 | 65.7 | ± | 4.2 |  |  |  | 368 | 224 | | 8 | 22.7 | ± | 1.6 |  |  |
|  | - | 129 | 359 | 64 | 67.3 | ± | 3.7 | 0.976 | 0.360 |  | 190 | 193 | | 10 | 32.2 | ± | 2.5 | 0.705 | <0.0001 |
|  |  |  |  |  |  |  |  |  |  |  |  |  | |  |  |  |  |  |  |
| **Reference** |  |  | *LEU2-LYS2* |  |  |  |  |  |  |  |  | *LEU2-LYS2* | |  |  |  |  |  |  |
|  |  |  | *LYS2-ADE2* |  |  |  |  |  |  |  |  | *ADE2-HIS3* | |  |  |  |  |  |  |
|  |  | PD | TT | NPD | cM |  | SE | ratio | p value |  | PD | TT | | NPD | cM |  | SE | ratio | p value |
|  | CO |  |  |  |  |  |  |  |  |  |  |  | |  |  |  |  |  |  |
| wild-type | + | 507 | 65 | 0 | 5.7 | ± | 0.7 |  |  |  | 186 | 378 | | 8 | 37.2 | ± | 1.6 |  |  |
|  | - | 296 | 198 | 2 | 21.2 | ± | 1.4 | 0.269 | <0.0001 |  | 157 | 331 | | 8 | 38.2 | ± | 1.8 | 0.974 | 0.843 |
|  |  |  |  |  |  |  |  |  |  |  |  |  | |  |  |  |  |  |  |
| *pch2*∆ | + | 418 | 176 | 5 | 17.2 | ± | 1.4 |  |  |  | 148 | 385 | | 60 | 62.8 | ± | 3.4 |  |  |
|  | - | 225 | 166 | 2 | 22.7 | ± | 1.6 | 0.758 | <0.0001 |  | 93 | 240 | | 52 | 71.7 | ± | 4.7 | 0.876 | 0.043 |
|  |  |  |  |  |  |  |  |  |  |  |  |  | |  |  |  |  |  |  |
|  |  |  |  |  |  |  |  |  |  |  |  |  | |  |  |  |  |  |  |
|  |  |  |  |  |  |  |  |  |  |  |  |  | |  |  |  |  |  |  |
|  |  |  |  |  |  |  |  |  |  |  |  |  | |  |  |  |  |  |  |
| **Reference** |  |  | *LYS2-ADE2* |  |  |  |  |  |  |  |  | *LYS2-ADE2* | |  |  |  |  |  |  |
|  |  |  | *URA3-LEU2* |  |  |  |  |  |  |  |  | *LEU2-LYS2* | |  |  |  |  |  |  |
|  |  | PD | TT | NPD | cM |  | SE | ratio | p value |  | PD | TT | | NPD | cM |  | SE | ratio | p value |
|  | CO |  |  |  |  |  |  |  |  |  |  |  | |  |  |  |  |  |  |
| wild-type | + | 150 | 115 | 0 | 21.7 | ± | 1.5 |  |  |  | 200 | 64 | | 1 | 13.2 | ± | 2.0 |  |  |
|  | - | 457 | 351 | 5 | 23.1 | ± | 1.2 | 0.939 | 0.195 |  | 396 | 505 | | 2 | 32.2 | ± | 1.0 | 0.410 | <0.0001 |
|  |  |  |  |  |  |  |  |  |  |  |  |  | |  |  |  |  |  |  |
| *pch2*∆ | + | 202 | 142 | 5 | 24.6 | ± | 2.2 |  |  |  | 168 | 169 | | 12 | 34.5 | ± | 3.0 |  |  |
|  | - | 353 | 275 | 13 | 27.5 | ± | 1.8 | 0.895 | 0.454 |  | 225 | 391 | | 27 | 43.0 | ± | 2.3 | 0.802 | <0.0001 |
|  |  |  |  |  |  |  |  |  |  |  |  |  | |  |  |  |  |  |  |
| **Reference** |  |  | *LYS2-ADE2* |  |  |  |  |  |  |  |  | *ADE2-HIS3* | |  |  |  |  |  |  |
|  |  |  | *ADE2-HIS3* |  |  |  |  |  |  |  |  | *URA3-LEU2* | |  |  |  |  |  |  |
|  |  | PD | TT | NPD | cM |  | SE | ratio | p value |  | PD | TT | | NPD | cM |  | SE | ratio | p value |
|  | CO |  |  |  |  |  |  |  |  |  |  |  | |  |  |  |  |  |  |
| wild-type | + | 107 | 153 | 5 | 34.5 | ± | 2.7 |  |  |  | 427 | 294 | | 4 | 21.9 | ± | 1.2 |  |  |
|  | - | 236 | 556 | 11 | 38.7 | ± | 1.4 | 0.891 | 0.0004 |  | 180 | 160 | | 1 | 24.5 | ± | 1.6 | 0.894 | 0.002 |
|  |  |  |  |  |  |  |  |  |  |  |  |  | |  |  |  |  |  |  |
| *pch2*∆ | + | 87 | 219 | 39 | 65.7 | ± | 4.7 |  |  |  | 423 | 310 | | 10 | 24.9 | ± | 1.5 |  |  |
|  | - | 154 | 413 | 74 | 66.9 | ± | 3.4 | 0.982 | 0.875 |  | 129 | 106 | | 8 | 31.7 | ± | 3.4 | 0.785 | 0.001 |
|  |  |  |  |  |  |  |  |  |  |  |  |  | |  |  |  |  |  |  |
| **Reference** |  |  | *ADE2-HIS3* |  |  |  |  |  |  |  |  | *ADE2-HIS3* | |  |  |  |  |  |  |
|  |  |  | *LEU2-LYS2* |  |  |  |  |  |  |  |  | *LYS2-ADE2* | |  |  |  |  |  |  |
|  |  | PD | TT | NPD | cM |  | SE | ratio | p value |  | PD | TT | | NPD | cM |  | SE | ratio | p value |
|  | CO |  |  |  |  |  |  |  |  |  |  |  | |  |  |  |  |  |  |
| wild-type | + | 339 | 384 | 2 | 27.3 | ± | 1.0 |  |  |  | 567 | 156 | | 2 | 11.6 | ± | 0.9 |  |  |
|  | - | 157 | 185 | 1 | 27.8 | ± | 2.0 | 0.982 | 0.867 |  | 236 | 102 | | 0 | 15.6 | ± | 1.3 | 0.744 | <0.0001 |
|  |  |  |  |  |  |  |  |  |  |  |  |  | |  |  |  |  |  |  |
| *pch2*∆ | + | 292 | 418 | 27 | 39.4 | ± | 2.1 |  |  |  | 487 | 255 | | 3 | 18.3 | ± | 1.1 |  |  |
|  | - | 93 | 137 | 11 | 42.1 | ± | 4.0 | 0.936 | 0.447 |  | 154 | 83 | | 4 | 22.2 | ± | 2.8 | 0.824 | 0.005 |
|  |  |  |  |  |  |  |  |  |  |  |  |  | |  |  |  |  |  |  |

|  |  |  |  |  |  |  |  |  | **Chromosome III** | | | |  |  |  |  |  |  |
| --- | --- | --- | --- | --- | --- | --- | --- | --- | --- | --- | --- | --- | --- | --- | --- | --- | --- | --- |
|  |  |  |  |  |  |  |  |  |  |  |  |  |  |  |  |  |  |  |
| **Reference** |  |  | *HIS4-LEU2* |  |  |  |  |  |  |  |  | *HIS4-LEU2* |  |  |  |  |  |  |
|  |  |  | *LEU2-CEN3* |  |  |  |  |  |  |  |  | *CEN3-MAT* |  |  |  |  |  |  |
|  |  | PD | TT | NPD | cM |  | SE | ratio | p value |  | PD | TT | NPD | cM |  | SE | ratio | p value |
|  | CO |  |  |  |  |  |  |  |  |  |  |  |  |  |  |  |  |  |
| wild-type | + | 140 | 5 | 0 | 1.7 | ± | 0.8 |  |  |  | 111 | 32 | 1 | 13.2 | ± | 2.6 |  |  |
|  | - | 349 | 63 | 0 | 7.7 | ± | 0.9 | 0.221 | <0.0001 |  | 284 | 128 | 0 | 15.5 | ± | 1.1 | 0.852 | <0.0001 |
|  |  |  |  |  |  |  |  |  |  |  |  |  |  |  |  |  |  |  |
| *pch2*∆ | + | 139 | 14 | 0 | 4.6 | ± | 1.2 |  |  |  | 107 | 44 | 0 | 14.6 | ± | 1.9 |  |  |
|  | - | 345 | 81 | 0 | 9.5 | ± | 1.0 | 0.484 | 0.003 |  | 310 | 109 | 1 | 13.7 | ± | 1.3 | 1.07 | 0.480 |
|  |  |  |  |  |  |  |  |  |  |  |  |  |  |  |  |  |  |  |
| *spo11-HA* | + | 89 | 8 | 0 | 4.1 | ± | 1.4 |  |  |  | 77 | 18 | 1 | 12.5 | ± | 3.6 |  |  |
|  | - | 335 | 76 | 0 | 9.3 | ± | 1.0 | 0.441 | 0.017 |  | 310 | 95 | 4 | 14.6 | ± | 1.8 | 0.856 | 0.567 |
|  |  |  |  |  |  |  |  |  |  |  |  |  |  |  |  |  |  |  |
| *pch2∆ spo11-HA* | + | 90 | 11 | 0 | 5.5 | ± | 1.6 |  |  |  | 93 | 8 | 0 | 4.0 | ± | 1.3 |  |  |
|  | - | 353 | 82 | 2 | 10.8 | ± | 1.3 | 0.509 | 0.058 |  | 378 | 58 | 0 | 6.7 | ± | 0.8 | 0.597 | 0.234 |
|  |  |  |  |  |  |  |  |  |  |  |  |  |  |  |  |  |  |  |
| **Reference** |  |  | *LEU2-CEN3* |  |  |  |  |  |  |  |  | *LEU2-CEN3* |  |  |  |  |  |  |
|  |  |  | *HIS4-LEU2* |  |  |  |  |  |  |  |  | *CEN3-MAT* |  |  |  |  |  |  |
|  |  | PD | TT | NPD | cM |  | SE | ratio | p value |  | PD | TT | NPD | cM |  | SE | ratio | p value |
|  | CO |  |  |  |  |  |  |  |  |  |  |  |  |  |  |  |  |  |
| wild-type | + | 63 | 5 | 0 | 3.7 | ± | 1.6 |  |  |  | 53 | 17 | 0 | 12.1 | ± | 2.6 |  |  |
|  | - | 349 | 139 | 1 | 14.8 | ± | 1.2 | 0.25 | <0.0001 |  | 350 | 147 | 1 | 15.4 | ± | 1.2 | 0.786 | 0.533 |
|  |  |  |  |  |  |  |  |  |  |  |  |  |  |  |  |  |  |  |
| *pch2*∆ | + | 81 | 14 | 0 | 7.4 | ± | 1.8 |  |  |  | 68 | 31 | 0 | 15.7 | ± | 2.3 |  |  |
|  | - | 345 | 136 | 3 | 15.9 | ± | 1.4 | 0.465 | 0.004 |  | 366 | 128 | 1 | 13.5 | ± | 1.1 | 1.16 | 0.398 |
|  |  |  |  |  |  |  |  |  |  |  |  |  |  |  |  |  |  |  |
| *spo11-HA* | + | 76 | 8 | 0 | 4.8 | ± | 1.6 |  |  |  | 69 | 15 | 1 | 12.4 | ± | 4.0 |  |  |
|  | - | 335 | 88 | 1 | 11.1 | ± | 1.2 | 0.432 | 0.017 |  | 324 | 98 | 4 | 14.3 | ± | 1.7 | 0.867 | 0.475 |
|  |  |  |  |  |  |  |  |  |  |  |  |  |  |  |  |  |  |  |
| *pch2∆ spo11-HA* | + | 84 | 11 | 0 | 5.8 | ± | 1.6 |  |  |  | 81 | 17 | 0 | 8.7 | ± | 1.9 |  |  |
|  | - | 353 | 89 | 1 | 10.7 | ± | 1.2 | 0.542 | 0.069 |  | 401 | 50 | 0 | 5.5 | ± | 0.7 | 1.58 | 0.183 |
|  |  |  |  |  |  |  |  |  |  |  |  |  |  |  |  |  |  |  |
| **Reference** |  |  | *CEN3-MAT* |  |  |  |  |  |  |  |  | *CEN3-MAT* |  |  |  |  |  |  |
|  |  |  | *HIS4-LEU2* |  |  |  |  |  |  |  |  | *LEU2-CEN3* |  |  |  |  |  |  |
|  |  | PD | TT | NPD | cM |  | SE | ratio | p value |  | PD | TT | NPD | cM |  | SE | ratio | p value |
|  | CO |  |  |  |  |  |  |  |  |  |  |  |  |  |  |  |  |  |
| wild-type | + | 128 | 33 | 0 | 10.3 | ± | 1.6 |  |  |  | 148 | 17 | 0 | 5.2 | ± | 1.2 |  |  |
|  | - | 284 | 110 | 1 | 14.7+ | ± | 1.3 | 0.701 | 0.064 |  | 350 | 53 | 0 | 6.6 | ± | 0.8 | 0.787 | 0.534 |
|  |  |  |  |  |  |  |  |  |  |  |  |  |  |  |  |  |  |  |
| *pch2*∆ | + | 110 | 43 | 1 | 15.9 | ± | 2.6 |  |  |  | 129 | 31 | 0 | 9.7 | ± | 1.6 |  |  |
|  | - | 310 | 105 | 2 | 14.0 | ± | 1.4 | 1.14 | 0.703 |  | 366 | 68 | 0 | 7.8 | ± | 0.9 | 1.24 | 0.456 |
|  |  |  |  |  |  |  |  |  |  |  |  |  |  |  |  |  |  |  |
| *spo11-HA* | + | 99 | 19 | 0 | 8.1 | ± | 1.7 |  |  |  | 102 | 16 | 0 | 6.8 | ± | 1.6 |  |  |
|  | - | 310 | 76 | 1 | 10.6 | ± | 1.3 | 0.764 | 0.445 |  | 324 | 69 | 0 | 8.8 | ± | 1.0 | 0.773 | 0.499 |
|  |  |  |  |  |  |  |  |  |  |  |  |  |  |  |  |  |  |  |
| *pch2∆ spo11-HA* | + | 58 | 8 | 0 | 6.1 | ± | 2.0 |  |  |  | 50 | 17 | 0 | 12.7 | ± | 2.7 |  |  |
|  | - | 378 | 92 | 1 | 10.4 | ± | 1.1 | 0.587 | 0.235 |  | 401 | 79 | 2 | 9.4 | ± | 1.2 | 1.35 | 0.136 |
|  |  |  |  |  |  |  |  |  |  |  |  |  |  |  |  |  |  |  |
|  |  |  |  |  |  |  |  |  | **Chromosome VIII** | | | | |  |  |  |  |  |
| **Reference** |  |  | *CEN8-THR1* |  |  |  |  |  |  |  |  | *THR1-CUP1* |  |  |  |  |  |  |
|  |  |  | *THR1-CUP1* |  |  |  |  |  |  |  |  | *CEN8-THR1* |  |  |  |  |  |  |
|  |  | PD | TT | NPD | cM |  | SE | ratio | p value |  | PD | TT | NPD | cM |  | SE | ratio | p value |
|  | CO |  |  |  |  |  |  |  |  |  |  |  |  |  |  |  |  |  |
| wild-type | + | 154 | 67 | 0 | 15.2 | ± | 1.6 |  |  |  | 194 | 67 | 0 | 12.8 | ± | 1.4 |  |  |
|  | - | 123 | 193 | 1 | 31.4 | ± | 1.6 | 0.484 | <0.0001 |  | 123 | 152 | 2 | 29.6 | ± | 2.0 | 0.432 | <0.0001 |
|  |  |  |  |  |  |  |  |  |  |  |  |  |  |  |  |  |  |  |
| *pch2*∆ | + | 108 | 113 | 12 | 39.7 | ± | 4.3 |  |  |  | 212 | 122 | 3 | 20.8 | ± | 1.9 |  |  |
|  | - | 80 | 193 | 19 | 52.6 | ± | 4.0 | 0.755 | <0.0001 |  | 80 | 104 | 4 | 34.0 | ± | 3.4 | 0.612 | <0.0001 |
|  |  |  |  |  |  |  |  |  |  |  |  |  |  |  |  |  |  |  |
| *spo11-HA* | + | 112 | 85 | 0 | 21.6 | ± | 1.8 |  |  |  | 234 | 85 | 0 | 13.3 | ± | 1.2 |  |  |
|  | - | 74 | 227 | 7 | 43.7 | ± | 2.5 | 0.494 | <0.0001 |  | 74 | 109 | 3 | 34.1 | ± | 3.1 | 0.39 | <0.0001 |
|  |  |  |  |  |  |  |  |  |  |  |  |  |  |  |  |  |  |  |
| *pch2∆ spo11-HA* | + | 88 | 67 | 9 | 36.9 | ± | 5.3 |  |  |  | 236 | 75 | 1 | 13.0 | ± | 1.5 |  |  |
|  | - | 139 | 225 | 11 | 38.8 | ± | 2.7 | 0.951 | <0.0001 |  | 139 | 85 | 3 | 22.7 | ± | 2.7 | 0.573 | <0.0001 |

|  |  |  |  |  |  |  |  |  | **Chromosome VII** | | | | |  |  |  |  |  |
| --- | --- | --- | --- | --- | --- | --- | --- | --- | --- | --- | --- | --- | --- | --- | --- | --- | --- | --- |
| **Reference** |  |  | *TRP5-CYH2* |  |  |  |  |  |  |  |  | *TRP5-CYH2* |  |  |  |  |  |  |
|  |  |  | *CYH2-MET13* |  |  |  |  |  |  |  |  | *MET13-LYS5* |  |  |  |  |  |  |
|  |  | PD | TT | NPD | cM |  | SE | ratio | p value |  | PD | TT | NPD | cM |  | SE | ratio | p value |
|  | CO |  |  |  |  |  |  |  |  |  |  |  |  |  |  |  |  |  |
| wild-type | + | 295 | 59 | 0 | 8.3 | ± | 1.0 |  |  |  | 225 | 118 | 3 | 19.7 | ± | 1.9 |  |  |
|  | - | 154 | 44 | 0 | 11.1 | ± | 1.5 | 0.748 | 0.034 |  | 109 | 87 | 1 | 23.6 | ± | 2.3 | 0.835 | 0.001 |
|  |  |  |  |  |  |  |  |  |  |  |  |  |  |  |  |  |  |  |
| *pch2*∆ | + | 275 | 116 | 5 | 18.4 | ± | 2.0 |  |  |  | 193 | 183 | 12 | 32.9 | ± | 2.7 |  |  |
|  | - | 93 | 38 | 0 | 14.5 | ± | 2.0 | 1.27 | 0.187 |  | 71 | 53 | 5 | 32.2 | ± | 5.2 | 1.02 | 0.049 |
|  |  |  |  |  |  |  |  |  |  |  |  |  |  |  |  |  |  |  |
| *spo11-HA* | + | 265 | 63 | 0 | 9.6 | ± | 1.1 |  |  |  | 187 | 140 | 0 | 21.4 | ± | 1.4 |  |  |
|  | - | 110 | 39 | 0 | 13.1 | ± | 1.8 | 0.733 | 0.012 |  | 85 | 63 | 1 | 23.2 | ± | 2.8 | 0.922 | 0.110 |
|  |  |  |  |  |  |  |  |  |  |  |  |  |  |  |  |  |  |  |
| *pch2∆ spo11-HA* | + | 297 | 54 | 1 | 8.5 | ± | 1.3 |  |  |  | 235 | 109 | 6 | 20.7 | ± | 2.3 |  |  |
|  | - | 133 | 28 | 0 | 8.7 | ± | 1.5 | 0.977 | 0.497 |  | 105 | 51 | 5 | 25.2 | ± | 4.3 | 0.821 | 0.242 |
|  |  |  |  |  |  |  |  |  |  |  |  |  |  |  |  |  |  |  |
| **Reference** |  |  | *CYH2-MET13* |  |  |  |  |  |  |  |  | *CYH2-MET13* |  |  |  |  |  |  |
|  |  |  | *TRP5-CYH2* |  |  |  |  |  |  |  |  | *MET13-LYS5* |  |  |  |  |  |  |
|  |  | PD | TT | NPD | cM |  | SE | ratio | p value |  | PD | TT | NPD | cM |  | SE | ratio | p value |
|  | CO |  |  |  |  |  |  |  |  |  |  |  |  |  |  |  |  |  |
| wild-type | + | 44 | 57 | 2 | 33.5 | ± | 4.4 |  |  |  | 82 | 20 | 0 | 9.8 | ± | 2.0 |  |  |
|  | - | 154 | 286 | 9 | 37.9 | ± | 2.1 | 0.884 | 0.206 |  | 253 | 187 | 5 | 24.4 | ± | 1.8 | 0.402 | <0.0001 |
|  |  |  |  |  |  |  |  |  |  |  |  |  |  |  |  |  |  |  |
| *pch2*∆ | + | 38 | 108 | 13 | 58.5 | ± | 6.0 |  |  |  | 89 | 65 | 3 | 26.4 | ± | 3.6 |  |  |
|  | - | 93 | 227 | 48 | 70.0 | ± | 4.8 | 0.836 | 0.109 |  | 180 | 174 | 14 | 35.1 | ± | 3.0 | 0.752 | 0.088 |
|  |  |  |  |  |  |  |  |  |  |  |  |  |  |  |  |  |  |  |
| *spo11-HA* | + | 39 | 61 | 2 | 35.8 | ± | 4.4 |  |  |  | 85 | 17 | 0 | 8.3 | ± | 1.9 |  |  |
|  | - | 110 | 245 | 20 | 48.7 | ± | 3.3 | 0.735 | 0.053 |  | 189 | 186 | 1 | 25.5 | ± | 1.5 | 0.325 | <0.0001 |
|  |  |  |  |  |  |  |  |  |  |  |  |  |  |  |  |  |  |  |
| *pch2∆ spo11-HA* | + | 28 | 47 | 8 | 57.2 | ± | 9.1 |  |  |  | 58 | 24 | 1 | 18.1 | ± | 4.2 |  |  |
|  | - | 133 | 266 | 31 | 52.6 | ± | 3.5 | 1.09 | 0.550 |  | 285 | 136 | 10 | 22.7 | ± | 2.3 | 0.797 | 0.637 |
|  |  |  |  |  |  |  |  |  |  |  |  |  |  |  |  |  |  |  |
| **Reference** |  |  | *MET13-LYS5* |  |  |  |  |  |  |  |  | *MET13-LYS5* |  |  |  |  |  |  |
|  |  |  | *TRP5-CYH2* |  |  |  |  |  |  |  |  | *CYH2-MET13* |  |  |  |  |  |  |
|  |  | PD | TT | NPD | cM |  | SE | ratio | p value |  | PD | TT | NPD | cM |  | SE | ratio | p value |
|  | CO |  |  |  |  |  |  |  |  |  |  |  |  |  |  |  |  |  |
| wild-type | + | 88 | 120 | 1 | 30.1 | ± | 2.1 |  |  |  | 192 | 20 | 0 | 4.7 | ± | 1.0 |  |  |
|  | - | 109 | 218 | 7 | 38.9 | ± | 2.5 | 0.774 | 0.004 |  | 253 | 82 | 0 | 12.2 | ± | 1.2 | 0.385 | <0.0001 |
|  |  |  |  |  |  |  |  |  |  |  |  |  |  |  |  |  |  |  |
| *pch2*∆ | + | 58 | 167 | 28 | 66.2 | ± | 5.3 |  |  |  | 188 | 65 | 3 | 16.2 | ± | 2.4 |  |  |
|  | - | 71 | 162 | 31 | 65.9 | ± | 5.4 | 1.00 | 0.282 |  | 180 | 87 | 2 | 18.4 | ± | 2.1 | 0.88 | 0.044 |
|  |  |  |  |  |  |  |  |  |  |  |  |  |  |  |  |  |  |  |
| *spo11-HA* | + | 64 | 128 | 12 | 49.0 | ± | 4.7 |  |  |  | 187 | 17 | 0 | 4.2 | ± | 1.0 |  |  |
|  | - | 85 | 177 | 10 | 43.6 | ± | 3.3 | 1.12 | 0.293 |  | 189 | 85 | 0 | 15.5 | ± | 1.4 | 0.271 | <0.0001 |
|  |  |  |  |  |  |  |  |  |  |  |  |  |  |  |  |  |  |  |
| *pch2∆ spo11-HA* | + | 56 | 103 | 12 | 51.2 | ± | 5.5 |  |  |  | 146 | 25 | 0 | 7.3 | ± | 1.4 |  |  |
|  | - | 105 | 208 | 27 | 54.4 | ± | 4.1 | 0.941 | 0.817 |  | 285 | 57 | 1 | 9.2 | ± | 1.3 | 0.793 | 0.465 |

**For Table S2:** Chromosome XV data were obtained from EAY background strains; chromosomes III, VII, and VIII data were obtained from NHY background strains. All pair-wise comparisons between adjacent intervals are shown. The top genetic interval listed in each box is the reference interval. All tetrads were divided into two classes: those with (CO +; i.e. NPD or TT) and those without (CO-; i.e. PD) an observable CO event within the reference interval using Mactetrad 6.9. The genetic size and standard error (SE) of the adjacent genetic interval (the lower listing at the top of the box) was then calculated for each class (CO+ and CO-) using the Stahl Laboratory Online Tools (http://molbio.uoregon.edu/~fstahl/). A ratio of the CO+/CO- class cM values was computed. Interference was considered significant if the CO+ and CO- classes were found to be significantly different via G-tests calculated using the spreadsheet available from The Online Handbook of Biological Statistics (http://udel.edu/~mcdonald/statintro.html).
