# Supplementary material for: The pch2Δ Mutation in Baker's Yeast Alters Meiotic Crossover Levels and Confers a Defect in Crossover Interference
Source: PLoS Genet. 2009 Jul 24;5(7):e1000571. doi: 10.1371/journal.pgen.1000571 (PMC2709914; doi:10.1371/journal.pgen.1000571)
Supplement: Table S3 — The recombination frequencies between the indicated markers and the number of parental and recombinant spores (as calculated by RANA software; Argueso et al. [11]) in the EAY strain background are shown. p values for G-tests comparing the recombinant and parental spore numbers for all mutant combinations were calculated using the spreadsheet available from The Online Handbook of Biological Statistics (http://udel.edu/~mcdonald/statintro.html). (0.08 MB DOC) [file pgen.1000571.s004.doc]

**Table S3. Genetic recombination frequencies in spores.**

| **Chromosome XV** | |  |  |  |  |  |  |  |  |  |
| --- | --- | --- | --- | --- | --- | --- | --- | --- | --- | --- |
|  |  |  |  |  |  |  | **p values** |  |  |  |
|  | **Total Spores** | **Recombinant** | **Parental** | **% Recombinant** |  | **to wild-type** | **to *pch2*∆** | **to *msh5∆*** | **to *pch2∆ msh5∆*** | **to *mms4∆*** |
| ***URA3-LEU2*** |  |  |  |  |  |  |  |  |  |  |
| wild-type | 4644 | 1009 | 3635 | 21.7 |  |  |  |  |  |  |
| *pch2*∆ | 4538 | 1067 | 3471 | 23.5 |  | 0.0039 |  |  |  |  |
| *msh5∆* | 5674 | 322 | 5352 | 5.7 |  | <0.0001 | <0.0001 |  |  |  |
| *pch2∆ msh5∆* | 859 | 83 | 776 | 9.7 |  | <0.0001 | <0.0001 | <0.0001 |  |  |
| *mms4*∆ | 2732 | 505 | 2227 | 18.5 |  | <0.0001 | <0.0001 | <0.0001 | <0.0001 |  |
| *mms4∆ pch2*∆ | 591 | 98 | 49 | 16.6 |  | 0.0017 | <0.0001 | <0.0001 | <0.0001 | 0.23 |
|  |  |  |  |  |  |  |  |  |  |  |
| ***LEU2-LYS2*** |  |  |  |  |  |  |  |  |  |  |
| wild-type | 4644 | 1256 | 3388 | 27.0 |  |  |  |  |  |  |
| *pch2*∆ | 4538 | 1458 | 3080 | 32.1 |  | <0.0001 |  |  |  |  |
| *msh5∆* | 5674 | 627 | 5047 | 11.1 |  | <0.0001 | <0.0001 |  |  |  |
| *pch2∆ msh5∆* | 859 | 80 | 779 | 9.3 |  | <0.0001 | <0.0001 | 0.096 |  |  |
| *mms4*∆ | 2732 | 651 | 2081 | 23.8 |  | 0.0001 | <0.0001 | <0.0001 | <0.0001 |  |
| *mms4∆ pch2*∆ | 591 | 166 | 425 | 28.1 |  | 0.570 | 0.033 | <0.0001 | <0.0001 | 0.017 |
|  |  |  |  |  |  |  |  |  |  |  |
| ***LYS2-ADE2*** |  |  |  |  |  |  |  |  |  |  |
| wild-type | 4644 | 592 | 4052 | 12.7 |  |  |  |  |  |  |
| *pch2*∆ | 4538 | 828 | 3710 | 18.2 |  | <0.0001 |  |  |  |  |
| *msh5∆* | 5674 | 265 | 5409 | 4.7 |  | <0.0001 | <0.0001 |  |  |  |
| *pch2∆ msh5∆* | 859 | 51 | 808 | 5.9 |  | <0.0001 | <0.0001 | 0.091 |  |  |
| *mms4*∆ | 2732 | 285 | 2447 | 10.4 |  | 0.0002 | <0.0001 | <0.0001 | <0.0001 |  |
| *mms4∆ pch2*∆ | 591 | 82 | 509 | 13.9 |  | 0.42 | 0.0044 | <0.0001 | <0.0001 | 0.0088 |
|  |  |  |  |  |  |  |  |  |  |  |
| ***ADE2-HIS3*** |  |  |  |  |  |  |  |  |  |  |
| wild-type | 4644 | 1611 | 3033 | 34.7 |  |  |  |  |  |  |
| *pch2*∆ | 4538 | 1978 | 2560 | 43.6 |  | <0.0001 |  |  |  |  |
| *msh5∆* | 5674 | 877 | 4797 | 15.5 |  | <0.0001 | <0.0001 |  |  |  |
| *pch2∆ msh5∆* | 859 | 202 | 657 | 23.5 |  | <0.0001 | <0.0001 | <0.0001 |  |  |
| *mms4*∆ | 2732 | 809 | 1923 | 29.6 |  | <0.0001 | <0.0001 | <0.0001 | <0.0001 |  |
| *mms4∆ pch2*∆ | 591 | 210 | 381 | 35.5 |  | 0.67 | <0.0001 | <0.0001 | <0.0001 | 0.0019 |

**For Table S3:** The recombination frequencies between the indicated markers and the number of parental and recombinant spores (as calculated by RANA software; Argueso et al. [11]) in the EAY strain background are shown. p values for G-tests comparing the recombinant and parental spore numbers for all mutant combinations were calculated using the spreadsheet available from The Online Handbook of Biological Statistics (http://udel.edu/~mcdonald/statintro.html).
